# Genetic tools for advancement of *Synechococcus* sp. PCC 7002 as a cyanobacterial chassis

**DOI:** 10.1186/s12934-016-0584-6

**Published:** 2016-11-10

**Authors:** Anne M. Ruffing, Travis J. Jensen, Lucas M. Strickland

**Affiliations:** Department of Bioenergy and Defense Technologies, Sandia National Laboratories, P.O. Box 5800, MS 1413, Albuquerque, NM 87185-1413 USA

**Keywords:** Synechococcus, Synechococcus sp. PCC 7002, Synechococcus 7002, Cyanobacterial chassis, Cyanobacterial genetic engineering, Cyanobacterial host, Cyanobacterial cell factories

## Abstract

**Background:**

Successful implementation of modified cyanobacteria as hosts for industrial applications requires the development of a cyanobacterial chassis. The cyanobacterium *Synechococcus* sp. PCC 7002 embodies key attributes for an industrial host, including a fast growth rate and high salt, light, and temperature tolerances. This study addresses key limitations in the advancement of *Synechococcus* sp. PCC 7002 as an industrial chassis.

**Results:**

Tools for genome integration were developed and characterized, including several putative neutral sites for genome integration. The minimum homology arm length for genome integration in *Synechococcus* sp. PCC 7002 was determined to be approximately 250 bp. Three fluorescent protein reporters (hGFP, Ypet, and mOrange) were characterized for gene expression, microscopy, and flow cytometry applications in *Synechococcus* sp. PCC 7002. Of these three proteins, the yellow fluorescent protein (Ypet) had the best optical properties for minimal interference with the native photosynthetic pigments and for detection using standard microscopy and flow cytometry optics. Twenty-five native promoters were characterized as tools for recombinant gene expression in *Synechococcus* sp. PCC 7002 based on previous RNA-seq results. This characterization included comparisons of protein and mRNA levels as well as expression under both continuous and diurnal light conditions. Promoters A2520 and A2579 were found to have strong expression in *Synechococcus* sp. PCC 7002 while promoters A1930, A1961, A2531, and A2813 had moderate expression. Promoters A2520 and A2813 showed more than twofold increases in gene expression under light conditions compared to dark, suggesting these promoters may be useful tools for engineering diurnal regulation.

**Conclusions:**

The genome integration, fluorescent protein, and promoter tools developed in this study will help to advance *Synechococcus* sp. PCC 7002 as a cyanobacterial chassis. The long minimum homology arm length for *Synechococcus* sp. PCC 7002 genome integration indicates native exonuclease activity or a low efficiency of homologous recombination. Low correlation between transcript and protein levels in *Synechococcus* sp. PCC 7002 suggests that transcriptomic data are poor selection criteria for promoter tool development. Lastly, the conventional strategy of using promoters from photosynthetic operons as strong promoter tools is debunked, as promoters from hypothetical proteins (A2520 and A2579) were found to have much higher expression levels.

**Electronic supplementary material:**

The online version of this article (doi:10.1186/s12934-016-0584-6) contains supplementary material, which is available to authorized users.

## Background

With the current emphasis on renewable energy and sustainable chemical production, cyanobacteria have emerged as photoautotrophic hosts for biofuel and chemical synthesis applications. While metabolic engineering efforts have successfully demonstrated cyanobacterial-based production of a wide range of fuels and chemicals [1–17], cyanobacterial hosts have yet to achieve commercial success. Strain development efforts for cyanobacteria are often limited by the availability of characterized genetic tools and our insufficient understanding of cyanobacterial metabolism and regulation. Complicating these efforts is the fact that multiple, diverse cyanobacterial hosts are used. The most common cyanobacterial hosts for genetic modification include two freshwater hosts, *Synechocystis* sp. PCC 6803 [5, 9, 10, 15, 16] and *Synechococcus elongatus* PCC 7942 [2, 4, 12, 18], and a marine host, *Synechococcus* sp. PCC 7002 [3, 13, 17]. Genetic tools developed for one cyanobacterial host are often directly used in another [13, 17], and cellular processes studied in one cyanobacterium are frequently assumed to be similar, if not identical, in another cyanobacterium. While some tools and cellular processes may be universal to cyanobacteria or eubacteria, this generalization among cyanobacterial species may limit their advancement as industrial hosts. For example, *Escherichia coli* has been studied and developed as a host for over four decades, and although genetic and functional comparisons may be drawn to other species, recombinant genetic tools have been shown to function in a host-specific manner [19]. In order to achieve advancements similar to that achieved in *E. coli*, we recommend that metabolic engineers focus on a single cyanobacterial host or chassis.

*Synechococcus* sp. PCC 7002 is an ideal host for the development of a cyanobacterial chassis. It has a fast doubling time (~2.6 h) [20]. As a marine strain, *Synechococcus* sp. PCC 7002 growth does not require freshwater resources; this is a key requirement for the production of high quantity, low-value commodities like biofuels. The salt tolerance of *Synechococcus* sp. PCC 7002 also allows for growth in open raceway pond production systems, where the salt concentration of the growth medium will fluctuate with evaporation. The high temperature tolerance of *Synechococcus* sp. PCC 7002 also enables growth in photobioreactors, where temperatures often exceed 40 °C [21]. Lastly, modified *Synechococcus* sp. PCC 7002 has demonstrated enhanced production of free fatty acids compared to the freshwater cyanobacterial host *S. elongatus* PCC 7942, suggesting improved host tolerance for the production of lipophilic fuels [13]. Despite these advantageous properties, the paucity of genetic tools available for modifying *Synechococcus* sp. PCC 7002 restricts the advancement of this cyanobacterial chassis.

Foreign genes and even entire pathways are often ported into chassis organisms, requiring either plasmid-based expression or identification of a neutral site for genome integration. As genome integration is more stable and predictable compared to plasmid-based expression, this is often the preferred method for modification, particularly for industrial microbial strains. The *desB* site in the *Synechococcus* sp. PCC 7002 genome has historically been used as a ‘neutral’ integration site, for *desB* has been shown to function primarily under temperatures much lower than the optimal growth temperature (18 vs 34–38 °C) [22]. However, if *Synechococcus* sp. PCC 7002 is to be employed under realistic outdoor growth conditions, environmental temperatures are likely to reach the range in which *desB* is expressed. Therefore, true neutral integration sites are necessary to advance *Synechococcus* sp. PCC 7002 as a chassis organism. Several putative neutral integration sites have been identified in recent efforts, including the pseudogene *glpK* (SYNPCC7002_A2842) [23] and the genomic region between hypothetical protein genes (SYNPCC7002_A0935 and SYNPCC7002_A0936) [3], yet the neutrality of these sites remains to be verified. Additionally, the annotated pseudogene SYNPCC7002_A2842 was recently shown to be a functional gene; it was originally annotated as a pseudogene due to a frameshift in the DNA sequence that was later shown to be a sequencing error [24].

Reporters are essential tools for chassis development, as they allow for easy quantitation of gene expression, visualization of subcellular localization, and high throughput screening via fluorescence activated cell sorting (FACS). While fluorescent protein genes and the *luxAB* bioluminescence operon have been used as reporters for gene expression in *Synechococcus* sp. PCC 7002 [25–28], there are very few examples of reporters used for microscopy or FACS applications with this organism. Additionally, the influence of cyanobacterial photosynthetic pigments (phycobilisomes and chlorophyll-*a*) on the optical properties of these reporters has not been characterized. Competitive absorbance of the excitation source, re-absorbance of fluorescent or bioluminescent reporter emission, and signal interference from the photosynthetic pigments may affect the application of these tools in *Synechococcus* sp. PCC 7002 and other cyanobacteria compared to traditional bacterial hosts.

Lastly, the advancement of *Synechococcus* sp. PCC 7002 as a cyanobacterial chassis is hindered by the lack of available characterized expression tools. Traditionally, genetic modification efforts in *Synechococcus* sp. PCC 7002 have relied on a few promoters for expression, such as cyanobacterial promoters associated with photosynthesis or inducible *E. coli* promoters with poor control in cyanobacterial hosts [29]. Recent efforts from the Pfleger laboratory report the development of promoter tools for *Synechococcus* sp. PCC 7002 using a random mutant library of the *cpc* promoter from *Synechocystis* sp. PCC 6803 and components of the lac and tet repressor systems [26, 28]. While these studies provide orthogonal promoter tools with a wide-range of expression in *Synechococcus* sp. PCC 7002, very little is known regarding how these heterologous tools will integrate with the natural metabolism and regulation of this organism and how expression may vary across the growth phase and with diurnal cycling. Both circadian rhythm and light conditions have been shown to regulate gene expression in cyanobacteria [30], and this dynamic regulation will likely be an important design parameter in synthetic biology applications. Thus, tools that can interface with the natural metabolism and regulation of *Synechococcus* sp. PCC 7002 are lacking.

In this study, we address technical limitations to the advancement of *Synechococcus* sp. PCC 7002 as a cyanobacterial chassis. Several neutral integration sites were identified and tested for neutrality, and the effect of homology arm length on the efficiency of genome integration in *Synechococcus* sp. PCC 7002 was characterized. Three fluorescent protein reporters were shown to be useful for microscopy and FACS applications. Twenty-five native promoters from *Synechococcus* sp. PCC 7002 were cloned upstream of a fluorescent reporter. Characterization of these promoters across the growth cycle and under both continuous and diurnal light conditions allows these promoters to be used as tools for recombinant gene expression and also provides insight into native promoter strength and regulation. This promoter information will provide metabolic engineers with basic guidelines for selecting and designing promoters for use in this cyanobacterial host.

## Methods

### Materials

Chemicals were purchased from Sigma-Aldrich (Na_2_EDTA·2H_2_O, CaCl_2_·2H_2_O, KH_2_PO_4_, vitamin B_12_, ZnCl_2_, and spectinomycin sulfate), MP Biomedicals (FeCl_3_·6H_2_O, CuSO_4_·5H_2_O, and CoCl_2_·6H_2_O), Acros (Na_2_MoO_4_·2H_2_O), Amresco (NaOAc, 3M, pH 5.2), Fisher Chemical (MnCl_2_·4H_2_O and Na_2_S_2_O_3_), and Fisher BioReagents [NaCl, MgSO_4_·7H_2_O, KCl, NaNO_3_, Tris base, H_3_BO_3_, kanamycin monosulfate, SDS, chloroform, saturated phenol (pH 4.3), and absolute ethanol]. Enzymes, including Q5 DNA polymerase, Taq polymerase, DNA ligase, and restriction enzymes, were purchased from New England Biolabs. DNA isolations and purifications were performed using the Zyppy Plasmid Miniprep kit, DNA Clean & Concentrator, and Zymoclean Gel DNA Recovery kit from Zymo Research. Genomic DNA was isolated using the GenElute Bacterial Genomic DNA kit (Sigma-Aldrich). All other vendors are indicated in the subsequent methods sections.

### Cultivation and transformation conditions

Cultures were grown in A+ medium [31] with antibiotics as needed within a New Brunswick Innova 42R shaking incubator with photosynthetic light bank. The optimum growth temperature for *Synechococcus* sp. PCC 7002 (34 °C) was used [13], along with shaking at 150 rpm and an average of 60 µmol photons m^−2^ s^−1^ of continuous or 12 h:12 h diurnal illumination from alternating cool white and plant fluorescent lights. Cultures maintained on agar plates were re-streaked every month to maintain the culture, and DMSO (5%) freezer stocks were stored at −80 °C [32]. Cultures were transferred from agar plates to 16 mm glass test tubes containing 4 mL of A+ medium. This test tube culture was grown for 4–7 days and then transferred to 100 mL of A+ medium in baffled 500 mL glass Erlenmeyer flasks with straight-neck flask closure cap at a dilution of 100×. For fluorescence spectra, fluorescence microscopy, and flow cytometry measurements of the strains expressing fluorescent proteins, samples were taken during the linear growth phase (note: when culturing cyanobacteria under these conditions, there is a very short exponential growth phase followed by a linear, light limited growth phase). For the neutral site and promoter expression strains, cell growth and photosynthetic yield or fluorescence measurements were taken every 2 days from the 100 mL cultures. A beaker filled with ultrapure water was maintained within the incubator to minimize evaporative loss of the cultures over time.

Transformation of *Synechococcus* sp. PCC 7002 was conducted based on previous protocols [13, 33]. Briefly, *Synechococcus* sp. PCC 7002 was grown to the mid-linear growth phase and either concentrated or diluted to an optical density at 730 nm (OD_730_) of 1.0, which was determined to be the optimal cell density for transformation in this study. 1 mL of this culture was placed in a 16 mm glass test tube with plastic closure cap, and 0.5 µg of linearized DNA in ultrapure water was added to this culture. The culture was placed back in the incubator under the standard conditions described above. To measure transformation efficiency, 100 µL of the transformation culture was spread on A+ medium agar plates containing the appropriate concentration of antibiotic (50 µg/mL kanamycin monosulfate). The number of colony forming units (cfu) on the agar plates was counted to obtain the number of transformed cells per 100 µL. The transformation efficiency was calculated using:

$${\text{Transformation efficiency}} = \frac{{{\text{cfu }} \times {\text{dilution factor}}}}{\text{fmol DNA}} \times {\text{fraction of PCR positive colonies }}$$

where the fraction of PCR positive colonies was determined by re-plating 50 colonies from each transformation plate and screening for insertion of the kanamycin resistance cassette using the screening primers desBscF and desBscR (Additional file 1: Table S1).

### Strain and plasmid construction

All strains used and constructed in this study are listed in Table 1. Putative neutral site (NS) integration strains were constructed using linear PCR fragments for genome integration. The linear fragments include an antibiotic resistance cassette (SpR or KmR) flanked by 500 bp homology sequences from the putative NS. The three fragments were amplified using PCR with Q5 DNA polymerase and stitched together using overlap PCR. The spectinomycin resistance cassette was amplified from pAM2991 (S. Golden, [18]); the kanamycin resistance cassette was amplified from pSB [13]; and the homology fragments were amplified from isolated genomic DNA from *Synechococcus* sp. PCC 7002. Primers used to construct these fragments and the sequences of the linear fragments can be found in the Additional file 1: Table S1. The linear fragments were purified from DNA gels and used for transformation of *Synechococcus* sp. PCC 7002 as described above.

**Table 1:** Strains used and constructed in this study

Strain	Description	Source
*Escherichia coli* DH5α	*E. coli* strain used for molecular cloning	New England Biolabs
*Synechococcus* sp. PCC 7002	Marine cyanobacterium, wild type	American Type Culture Collection
ΔNS1	*Synechococcus* sp. PCC 7002 with spectinomycin resistance cassette integrated at putative neutral site 1	This study
ΔNS2	*Synechococcus* sp. PCC 7002 with kanamycin resistance cassette integrated at putative neutral site 2	This study
7002-hGFP	*Synechococcus* sp. PCC 7002 with hGFP, driven by P_rbc_, integrated at NS2	This study
7002-Ypet	*Synechococcus* sp. PCC 7002 with Ypet, driven by P_rbc_, integrated at NS2	This study
7002-mOrange	*Synechococcus* sp. PCC 7002 with mOrange, driven by P_rbc_, integrated at NS2	This study
A0047	*Synechococcus* sp. PCC 7002 with Ypet, driven by P_0047_, integrated at NS2	This study
A0255	*Synechococcus* sp. PCC 7002 with Ypet, driven by P_0255_, integrated at NS2	This study
A0304	*Synechococcus* sp. PCC 7002 with Ypet, driven by P_0304_, integrated at NS2	This study
A0318	*Synechococcus* sp. PCC 7002 with Ypet, driven by P_0318_, integrated at NS2	This study
A0670	*Synechococcus* sp. PCC 7002 with Ypet, driven by P_0670_, integrated at NS2	This study
A0740	*Synechococcus* sp. PCC 7002 with Ypet, driven by P_0740_, integrated at NS2	This study
A1173	*Synechococcus* sp. PCC 7002 with Ypet, driven by P_1173_, integrated at NS2	This study
A1181	*Synechococcus* sp. PCC 7002 with Ypet, driven by P_1181_, integrated at NS2	This study
A1731	*Synechococcus* sp. PCC 7002 with Ypet, driven by P_1731_, integrated at NS2	This study
A1929	*Synechococcus* sp. PCC 7002 with Ypet, driven by P_1929_, integrated at NS2	This study
A1930	*Synechococcus* sp. PCC 7002 with Ypet, driven by P_1930_, integrated at NS2	This study
A1961	*Synechococcus* sp. PCC 7002 with Ypet, driven by P_1961_, integrated at NS2	This study
A1962	*Synechococcus* sp. PCC 7002 with Ypet, driven by P_1962_, integrated at NS2	This study
A2062	*Synechococcus* sp. PCC 7002 with Ypet, driven by P_2062_, integrated at NS2	This study
A2127	*Synechococcus* sp. PCC 7002 with Ypet, driven by P_2127_, integrated at NS2	This study
A2165	*Synechococcus* sp. PCC 7002 with Ypet, driven by P_2165_, integrated at NS2	This study
A2210	*Synechococcus* sp. PCC 7002 with Ypet, driven by P_2210_, integrated at NS2	This study
A2520	*Synechococcus* sp. PCC 7002 with Ypet, driven by P_2520_, integrated at NS2	This study
A2531	*Synechococcus* sp. PCC 7002 with Ypet, driven by P_2531_, integrated at NS2	This study
A2579	*Synechococcus* sp. PCC 7002 with Ypet, driven by P_2579_, integrated at NS2	This study
A2595	*Synechococcus* sp. PCC 7002 with Ypet, driven by P_2595_, integrated at NS2	This study
A2596	*Synechococcus* sp. PCC 7002 with Ypet, driven by P_2596_, integrated at NS2	This study
A2663	*Synechococcus* sp. PCC 7002 with Ypet, driven by P_2663_, integrated at NS2	This study
A2813	*Synechococcus* sp. PCC 7002 with Ypet, driven by P_2813_, integrated at NS2	This study

Genome integration plasmids with varying lengths of homology arms (250, 500, 750, 1000, and 1250 bp) were constructed for integration at *desB* (Synpcc7002_A0158) in *Synechococcus* sp. PCC 7002. The knockout plasmid pSB was previously constructed for integration at *desB* with homology arms of 1000 bp flanking a kanamycin resistance cassette. These homology arms were extended to 1250 bp each and shortened to 750, 500, and 250 bp on each flanking region by amplifying these 5′ and 3′ fragments from *Synechococcus* sp. PCC 7002 gDNA using Q5 DNA polymerase and the primers listed in the Additional file 1: Table S1. The fragments and pSB were digested with *Sac*I for integration of the 5′ homology arm and with *Avr*II for integration of the 3′ homology arm. Successful ligation of the 5′ and 3′ homology arms was confirmed using PCR amplification. The resulting plasmids, pSB1250, pSB, pSB750, pSB500, and pSB250, were linearized using SpeI digestion, followed by heat inactivation, and transformed into *Synechococcus* sp. PCC 7002 as described above.

Three fluorescent proteins, hGFP, Ypet, and mOrange, were selected for expression in *Synechococcus* sp. PCC 7002. The hybrid GFP (hGFP) sequence includes mutations from both a FACS optimized GFP variant [34] (Accession Number: U73901), EGFP (Clontech, Accession Number: U55762), and GFPmut2 [35]. All fluorescent protein genes were codon optimized for expression in *Synechococcus* sp. PCC 7002 using the online codon optimization tool from Integrated DNA Technologies (IDT). The promoter and terminator regions of the native *rbc* operon flank each of the fluorescent proteins, and the 5′ homology arm (500 bp) from NS2 was placed upstream of the *rbc* promoter. The entire fragment, containing the 5′ NS2 homology arm (NS2_5′), *rbc* promoter (P_rbc_), codon optimized fluorescent protein gene (FP), and *rbc* terminator (T_rbc_), was synthesized for each construct using IDT’s gBlocks gene fragments. A kanamycin resistance cassette (KmR) and the 3′ homology arm for NS2 (NS2_3′) were amplified using primers KmRF and NS2_3R and inserted downstream of the *rbc* terminator in each cassette using overlap PCR (see primers in Additional file 1: Table S1). Each PCR amplified linear integration cassette (NS2_5′-P_rbc_-FP-T_rbc_-KmR-NS2_3′) was purified and transformed into *Synechococcus* sp. PCC 7002 as described above.

To construct the NS2 genome integration plasmid with Ypet expression from the *rbc* promoter, the *ypet* integration cassette (NS2_5′-P_rbc_-Ypet-T_rbc_-KmR-NS2_3′) was amplified using primers to insert *Sac*I and *Avr*II restriction sites at the 5′ and 3′ ends (Additional file 1: Table S1). This amplified PCR fragment and pSB were digested with *Sac*I and *Avr*II and ligated to produce pSBP_rbc_Ypet. To allow for exchange of the promoter region, P_rbc_ was removed from pSBP_rbc_Ypet, and *Kpn*I and *Nde*I restrictions sites were added upstream of *ypet* to yield pSBYpet (see Additional file 1: Table S1 for primers). For each of the 24 native *Synechococcus* sp. PCC 7002 loci, 500 bp upstream of the start codon was amplified, digested, and ligated to pSBYpet, yielding plasmids for genome integration at NS2 (Additional file 1: Table S2). The promoter expression plasmids were digested with SpeI and transformed into *Synechococcus* sp. PCC 7002 as described above.

### Spectroscopy measurements

To estimate cell concentration of the *Synechococcus* sp. PCC 7002 cultures, optical density (OD) was measured at 730 nm using a PerkinElmer Lambda Bio spectrophotometer. DNA concentration was measured using 2 µL of purified DNA and a Nanodrop 2000 spectrophotometer.

A Jasco J-815 CD spectrometer was used to measure the fluorescence excitation and emission spectra of the strains engineered to express fluorescent proteins. The optimum excitation wavelength for each fluorescent protein was determined from an excitation scan at the optimum emission wavelengths (520 nm for hGFP, 565 nm for Ypet, and 600 nm for mOrange), and the optimum emission wavelength for each fluorescent protein was determined from an emission scan with near-optimum excitation wavelengths (465 nm for hGFP, 485 nm for Ypet, and 515 for mOrange). For each scan, the following settings were used: data pitch = 0.1 nm, sensitivity = 900 volts, Ex bandwidth = 10 nm, Em bandwidth = 10 nm, scanning speed = 100 nm/min, accumulations = 4.

For the promoter expression strains, 200 µL of appropriately diluted culture were placed in a Corning clear bottom 96-well plate, and a BioTek Synergy H4 microplate reader measured optical density at 730 nm. The microplate reader was also used to measure Ypet fluorescence of the promoter expression strains from 200 µL of culture in Costar black bottom 96-well plates with 485/20 nm excitation, 528/20 nm emission detection, a gain of 120, and a read height of 5 mm. Samples that saturated the detector under these conditions were diluted with A+ medium until the fluorescence emission was within the range of detection. Normalized fluorescence readings for each promoter were calculated by using linear interpolation to determine fluorescence readings for culture ODs matching those previously used during acquisition of RNA-seq data [36] (OD_730_ = 0.4, 0.7, 1.0, 3.0, and 5.0).

### Fluorescence microscopy

An Olympus IX71 confocal fluorescence microscope with a 60×/1.42 oil objective was used to analyze the fluorescent protein expressing strains of *Synechococcus* sp. PCC 7002. The culture samples (1.5 mL) were centrifuged at 5000×*g* for 5 min, and the cell pellets were resuspended in approximately 50 µL of supernatant to concentrate the culture. A 10 µL aliquot of each culture was placed on a glass microscope slide, covered with a no. 1.0 cover slip, and sealed with nail polish. A Prior Scientific Lumen 200PRO fluorescence illumination system with a Sutter Instrument Lambda 10-3 filter wheel was used to excite the samples. The Chroma Chl LP filter cube (Em > 600 nm) with 484 nm excitation was used to detect chlorophyll-*a* (Chl-*a*) fluorescence; the Semrock GFP-3035B-OMF-ZERO (Em 520/35 nm) filter cube with 484 or 500 nm excitation was used to detect hGFP and Ypet, respectively; and the Olympus DSU-MRFPHQ (Em 597.5/55 nm) filter cube with 534 nm excitation was used to detect mOrange. SlideBook 6 software was used for image acquisition. The images were imported into ImageJ [37], upon which Chl-*a* fluorescence was colored red; fluorescent protein fluorescence was colored green; and scale bars were added.

### Flow cytometry

An Accuri C6 flow cytometer was used for analyzing the *Synechococcus* sp. PCC 7002 strains engineered with fluorescent proteins. The optimal flowrate for *Synechococcus* sp. PCC 7002 was determined to be medium speed (35 µL/min, 16 µm core size) based on the best correlation between hemocytometer and flow cytometer cell counts. A cutoff of 50,000 on FSC-H was set, and 20,000 events were recorded for each run. Each sample was diluted with A+ medium so that the number of events per second was less than 650, which was determined to be limit for accurate counting of *Synechococcus* sp. PCC 7002 cells.

### Quantitative reverse transcriptase PCR (qRT-PCR)

To measure *ypet* expression levels under 12:12 light:dark conditions, 30 mL samples were extracted from cultures 6 h after the lights turned on and 6 h after the lights turned off after 5 days of incubation under diurnal conditions. The samples were placed in 50 mL ice-chilled, conical tubes and centrifuged at 3900×*g* for 4 min at 4 °C. The supernatant was decanted, and the cell pellets were immediately frozen in liquid nitrogen and stored at −80 °C until RNA extraction. A hot acid phenol extraction method was used for RNA extraction, as described previously for *S. elongatus* PCC 7942 [12]. Any remaining DNA was removed from the RNA samples using the TURBO DNA-free kit (Ambion, Life Technologies). Isolated RNA was quantified using the Quant-iT RiboGreen RNA assay kit (Life Technologies) with fluorescence measured by a NanoDrop 3300 fluorospectrometer. Complementary DNA (cDNA) was synthesized using approximately 2 µg of RNA and a Superscript III First-Strand synthesis kit with random primers (Invitrogen, Life Technologies). Any remaining RNA was removed using RNase OUT, provided within the cDNA synthesis kit. The cDNA was diluted 10× and used as template with primers (200 µM final concentration) to amplify a 159 bp region within *ypet* (Additional file 1: Table S1) along with Power SYBR Green PCR Master Mix (Life Technologies) in an Applied Biosystems 7300 Real-Time PCR system for quantification. For relative quantification, *rnpA,* previously reported as a stable housekeeping gene for qPCR [38], was used as a reference gene with a 176 bp amplicon (see Additional file 1: Table S1 for primers). Three technical replicates were included for each sample along with no template and no reverse transcriptase controls. The three technical replicate C_T_ values were averaged, and the 2^−ΔΔCT^ method was used for relative quantification [39]. Two biological replicates were analyzed for each promoter expression strain, and the average of these biological replicates is reported along with the standard deviation.

## Results

### Genome integration tools for *Synechococcus* sp. PCC 7002

To identify neutral sites (NS) for genome integration in *Synechococcus* sp. PCC 7002, the genome sequence was analyzed to detect large regions within the genome with no predicted function or annotation. Only three such regions were found to be greater than 1 kb in the *Synechococcus* sp. PCC 7002 genome: nucleotides 963,217–964,242 between SYNPCC7002_A0932 and SYNPCC7002_A0933 (neutral site 1—NS1), nucleotides 1247,018–1248,056 between SYNPCC7002_A1202 and SYNPCC7002_A1203 (neutral site 2—NS2), and nucleotides 1,864,422–1,865,821 between SYNPCC7002_A1778 and SYNPCC7002_A1779 (neutral site 3—NS3). Genome integration fragments were designed for the first two putative neutral integration sites, using spectinomycin adenyltransferase (*aadA*), a spectinomycin resistance cassette, and neomycin phosphotransferase (*neo*), a kanamycin resistance cassette, flanked by 500 bp sequences homologous to NS1 and NS2, respectively. These genome integration fragments were used to construct ΔNS1 and ΔNS2 strains of *Synechococcus* sp. PCC 7002, as described in the Materials and Methods section. Under standard growth conditions (34 °C, 150 rpm, and 60 µmol photons m^−2^ s^−1^ of continuous light), ΔNS1 and ΔNS2 did not show any significant changes in growth or photosynthetic efficiency compared to the wild type (Table 2, two-tail p values >0.3), suggesting that NS1 and NS2 are neutral integration sites under these conditions.

**Table 2:** Physiological properties (linear growth rate and photosynthetic efficiency) of wild type *Synechococcus* sp. PCC 7002 and putative neutral site integration strains

Strain	Linear growth rate (OD_730_/h)	Photosynthetic efficiency (F_v_’/F_m_’)
*Synechococcus* sp. PCC 7002	0.0334 ± 0.0103	0.191 ± 0.0712
ΔNS1	0.0371 ± 0.0149	0.203 ± 0.0422
ΔNS2	0.0346 ± 0.00923	0.163 ± 0.0428

For each neutral site integration strain, six transformants were tested, and at least three biological replicate experiments were performed for each strain using standard growth conditions. Data are the averages of all biological replicates and all transformants with the standard deviation reported as error. A two sample t test assuming equal variances compared ΔNS1 and ΔNS2 to the wild type to confirm that these values are statistically similar (two-tail p values >0.05)

**Fig. 1:**
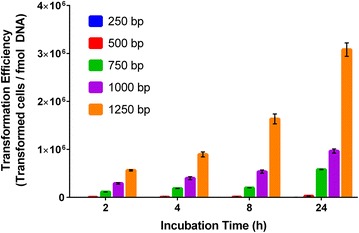
Transformation efficiency of *Synechococcus* sp. PCC 7002 as a function of incubation time with varying lengths of homology arms (250–1250 bp) for genome integration at *desB*

Another important consideration for genome integration is the required length of homology arms for efficient homologous recombination in *Synechococcus* sp. PCC 7002. Homology arms are the DNA regions homologous to the target site in the genome and flanking a selectable marker. We tested homology arms ranging from 250 bp to 1250 bp for integration of the kanamycin resistance cassette at *desB* in *Synechococcus* sp. PCC 7002. It is important to note that these homology arms were cloned into an integration plasmid which was linearized for transformation rather than PCR fragments which would be susceptible to exonuclease degradation. As expected, we found that the transformation efficiency of the integration plasmid increased with increasing length of the homology arms and time of incubation (Fig. 1). Under the conditions used for transformation of *Synechococcus* sp. PCC 7002 in this study, homology arms of 250 bp appear to be the minimum length required for successful transformation, as only 0–84 colonies were obtained with this integration plasmid across incubation times ranging from 2 to 24 h. The transformation efficiency calculation accounts for the fraction of colonies that have integration of the kanamycin resistance cassette, as confirmed by PCR screening of 50 colonies for each integration plasmid with three biological replicates. Integration plasmids with homology arms greater than 750 bp had a high percentage of colonies with confirmed integration, >86 ± 13%; while integration plasmids with homology arms of 500 and 250 bp had only 61 ± 17 and 34 ± 26% of colonies with positive PCR bands, respectively. Thus, targeted integration of the resistance cassette also increased with increasing length of the homology arms.

### Fluorescent protein reporters for gene expression, microscopy, and flow cytometry

Fluorescent protein reporters are often used in chassis organisms to analyze gene expression or to determine the subcellular localization of a recombinant protein by fusing the target protein with the reporter. The use of fluorescent protein reporters in cyanobacteria is complicated by absorbance and fluorescence of native photosynthetic pigments. Therefore, fluorescent protein reporters for *Synechococcus* sp. PCC 7002 were selected based on the absorbance and fluorescence spectra for this host. Three fluorescent protein genes were codon optimized for expression in *Synechococcus* sp. PCC 7002: a hybrid green fluorescent protein gene (*hgfp*), a yellow fluorescent protein gene (*ypet*), and an orange fluorescent protein gene (*morange*) [40]. The native *rbc* promoter and transcription terminator regions were placed upstream and downstream of each codon-optimized fluorescent protein gene; a kanamycin resistance cassette was placed downstream of the fluorescent protein operon; and 500 bp homology arms for genome integration at NS2 were placed as bookends for the entire insertion fragment. The resulting PCR fragments were used to generate engineered strains containing the three fluorescent protein reporters.

**Fig. 2:**
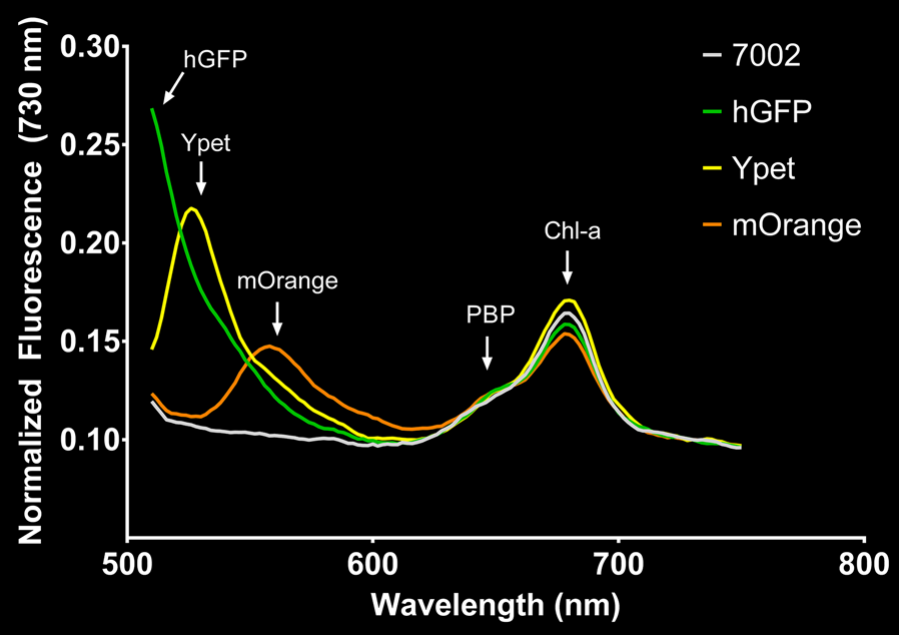
Fluorescence emission spectra of *Synechococcus* sp. PCC 7002 (wild type) and engineered strains expressing hGFP, Ypet, and mOrange with excitation at 488 nm

**Fig. 3:**
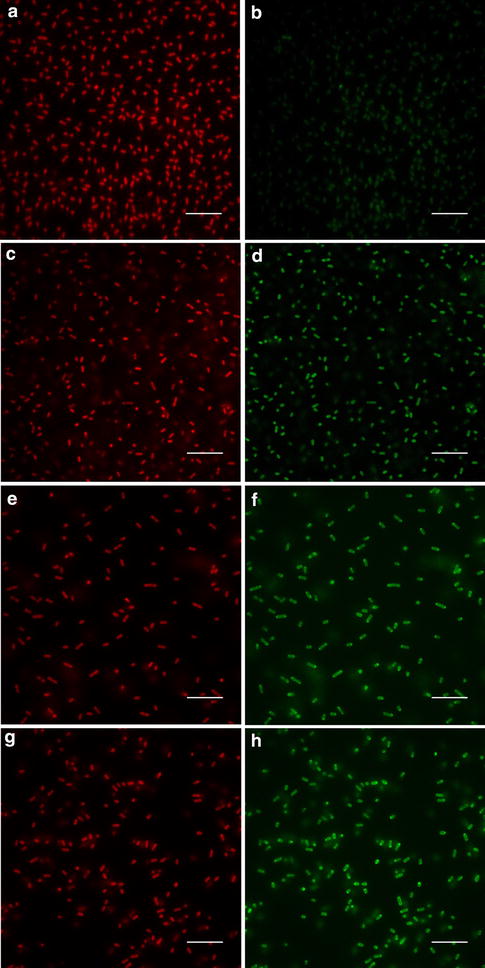
Fluorescence microscopy images of wild type *Synechococcus* sp. PCC 7002 (**a**, **b**) and engineered strains expressing hGFP (**c**, **d**), Ypet (**e**, **f**), and mOrange (**g**, **h**). Chlorophyll-*a* fluorescence (Ex 484/Em >600) is shown in **a**, **c**, **e**, and **g** while the recombinant fluorescent protein fluorescence (Ex 484/Em 520—GFP, Ex 500/Em 520—Ypet, and Ex 534/Em 597.6—mOrange) is shown in **b**, **d**, **f**, and **h**. *Scale bar* 10 µm

The excitation and emission peaks for each *Synechococcus* sp. PCC 7002 strain expressing a fluorescent protein were determined to be Ex 468–490 nm and Em 507 nm for hGFP, Ex 517 nm and Em 530 nm for Ypet, and Ex 545 nm and Em 560 nm for mOrange (Additional file 1: Figure S1). Using 488 nm excitation, the emission peaks from each of the fluorescent proteins were clearly distinguishable from the spectra of the wild type, but the fluorescence emission peak from hGFP showed some overlap with the shoulder of the fluorescence emission peak from the chlorophyll-*a* Soret band (Fig. 2). The engineered strains expressing fluorescent proteins were also analyzed using confocal fluorescence microscopy to determine the feasibility of utilizing these reporters for cellular imaging. As shown in Fig. 3, all three fluorescent proteins were visualized along with chlorophyll-*a* as a control. Lastly, the strains expressing fluorescent proteins were analyzed using flow cytometry to illustrate the feasibility of utilizing these reporters for FACS. Additional file 1: Figure S2 shows that the hGFP and Ypet expressing strains were readily distinguished from wild type using 488 nm excitation and a 533/30 nm emission filter (FL-1) for detection; however, the mOrange expressing strain was not clearly identified by the Accuri C6 flow cytometer’s 488 nm excitation and 533/30 nm (FL-1) or 585/40 nm (FL-2) detection filters. A flow cytometer equipped with laser excitation near 548 nm and an emission filter near 562 nm, the excitation and emission maxima of mOrange, should allow for detection and subsequent sorting of mOrange expressing strains.

### Native promoters as tools for recombinant gene expression

In order to select a representative sample of native promoters for cloning and characterization, RNA-seq data previously collected for *Synechococcus* sp. PCC 7002 at various cell concentrations throughout its growth profile (OD_730_ = 0.4, 0.7, 1.0, 3.0, and 5.0) [36] were used to select promoters of various strengths (10^−2^, 10^−3^, 10^−4^, and 10^−5^ counts/total counts) and expression patterns (constitutive, linear phase, and stationary phase). For each expression pattern, two promoters were selected for each strength, yielding 8 promoters for a given expression pattern. Overall, 24 native promoters from the RNA-seq data were analyzed along with the commonly used *rbc* promoter for comparison (Table 3). This promoter list includes several adjacent gene loci that were predicted by the Database of prOkaryotic OpeRons (DOOR) [41] to be independent operons containing similar functional protein genes (A1929/A1930 and A1961/A1962). For each promoter, a 500 bp sequence upstream of the target gene was cloned and placed upstream of *ypet* in pSYpet. After integration of each promoter-*ypet* fragment into NS2 of *Synehococcus* sp. PCC 7002, the promoter expression strains were analyzed for Ypet expression under both continuous (60 µmol photons m^−2^ s^−1^) and diurnal (12 h/12 h light/dark) conditions.

**Table 3:** Comparison of expression level and regulatory pattern for RNA-seq results [36] and Ypet fluorescence for native *Synechococcus* sp. PCC 7002 promoters

Promoter gene locus	Native gene locus function	Expression level		Regulatory pattern		Correlation coefficient (Ypet vs RNA-seq)
		Average RNA-seq (counts/total counts)	Average Ypet fluorescence (normalized to 7002)	RNA-seq (slope of counts/total counts vs. OD_730_)	Ypet fluorescence (slope of fluorescence vs. OD_730_)	
Synpcc7002_A0047	Hypothetical protein	2.17E−04	1.322 ± 0.116*	Stationary phase (9.68E−04)	Constitutive (−0.0192)	0.0967
Synpcc7002_A0255	UDP-N-acetyl-D-mannosaminuronic acid transferase	5.09E−05	1.073 ± 0.133	Stationary phase (1.63E−05)	Constitutive (−0.0265)	−0.722
Synpcc7002_A0304	Hypothetical protein	5.55E−05	1.038 ± 0.158	Linear phase (−1.77E−05)	Constitutive (−0.107)	0.715
Synpcc7002_A0318	Hypothetical protein	8.72E−04	1.137 ± 0.135*	Constitutive (5.75E−06)	Constitutive (0.196)	0.212
Synpcc7002_A0670	Hypothetical protein	2.16E−05	1.239 ± 0.442	Constitutive (5.72E−07)	Constitutive (0.178)	0.678
Synpcc7002_A0740	ATP synthase subunit I	1.04E−03	1.186 ± 0.0919*	Linear phase (−2.73E−04)	Constitutive (3.83E−04)	−0.628
Synpcc7002_A1173	Polyketide synthase	1.22E−03	1.087 ± 0.0914*	Constitutive (1.62E−05)	Constitutive (0.172)	0.252
Synpcc7002_A1181	ATPase AAA	2.58E−03	1.074 ± 0.0937	Stationary phase (9.41E−04)	Constitutive (−0.0108)	−0.0121
Synpcc7002_A1731	Hypothetical protein	6.03E−05	1.075 ± 0.0967	Constitutive (−8.49E−07)	Constitutive (0.0640)	0.181
Synpcc7002_A1798 (*rbc*)	Ribulose bisophosphate carboxylase, large subunit	4.77E−03	4.153 ± 2.17*	Linear phase (−6.24E−04)	Stationary phase (2.88)	−0.335
Synpcc7002_A1929	Allophycocyanin subunit beta	1.29E−02	1.046 ± 0.0866	Linear phase (−2.75E−03)	Constitutive (−0.0547)	0.800
Synpcc7002_A1930	Allophycocyanin subunit alpha	7.46E−03	2.338 ± 0.197*	Linear phase (−1.51E−03)	Constitutive (−0.107)	0.249
Synpcc7002_A1961	Photosystem I P700 chlorophyll-a apoprotein A1	1.95E−02	2.076 ± 0.237*	Constitutive (6.59E−04)	Stationary phase (0.744)	0.348
Synpcc7002_A1962	Photosystem I P700 chlorophyll-a apoprotein A2	2.61E−02	1.059 ± 0.126	Stationary phase (2.42E−03)	Constitutive (−0.0533)	0.256
Synpcc7002_A2062	Elongation factor G	5.33E−03	1.038 ± 0.00945*	Constitutive (3.95E−06)	Constitutive (0.0392)	−0.0315
Synpcc7002_A2127	Acetyl-CoA carboxylase biotin carboxylase subunit	3.62E−04	1.073 ± 0.0576*	Constitutive (1.64E−06)	Constitutive (0.0583)	0.457
Synpcc7002_A2165	Hypothetical protein	9.95E−05	1.205 ± 0.129*	Stationary phase (3.20E−05)	Constitutive (0.148)	0.960
Synpcc7002_A2210	C-phycocyanin subunit alpha	2.91E−02	1.129 ± 0.191	Linear phase (−4.85E−03)	Constitutive (0.163)	−0.907
Synpcc7002_A2520	Hypothetical protein	7.27E−05	31.88 ± 4.61*	Linear phase (−2.78E−05)	Stationary phase (35.4)	−0.954
Synpcc7002_A2531	Hypothetical protein	1.24E−02	2.340 ± 0.199*	Constitutive (−7.74E−04)	Constitutive (0.105)	−0.638
Synpcc7002_A2579	Hypothetical protein	6.80E−03	33.51 ± 6.28*	Linear phase (−2.79E−03)	Stationary phase (30.2)	−0.968
Synpcc7002_A2595	Hypothetical protein	9.20E−04	1.061 ± 0.107	Stationary phase (3.90E−04)	Constitutive (−0.0697)	−0.972
Synpcc7002_A2596	Hypothetical protein	2.77E−04	1.108 ± 0.0762*	Stationary phase (9.99E−05)	Constitutive (−0.0649)	−0.895
Synpcc7002_A2663	Bacterioferritin	2.87E−04	1.490 ± 0.169*	Linear phase (−6.99E05)	Constitutive (0.0140)	−0.116
Synpcc7002_A2813	S-layer protein	2.47E−02	3.486 ± 0.791*	Stationary phase (2.26E−03)	Constitutive (0.108)	−0.213

Only results for continuous light conditions are shown. Average Ypet fluorescence values having a change that is statistically significant (two-tail p value <0.05) compared to the wild type are indicated with an asterisk. Linear phase and stationary phase expression are defined as having more than a 30% change in relative expression. High positive slopes of fluorescence vs. OD730 indicate stationary phase expression, high negative slopes suggest linear phase expression, and intermediate slopes are consistent with constitutive expression

As the RNA-seq data was collected under continuous light conditions (250 µmol photons m^−2^ s^−1^) [36], the Ypet expression levels measured under continuous light in this study (60 µmol photons m^−2^ s^−1^) were used for comparison (Table 3). In general, the expression levels measured by Ypet fluorescence of the engineered strains were low; in fact, only 7 of the 25 promoters showed more than a 50% increase in Ypet fluorescence compared to the wild type. This includes moderate promoters, demonstrating an average 2- to 6-fold increase in Ypet fluorescence (P_A1798_, P_A1930_, P_A1961_, P_A2531_, and P_A2813_), and two strong promoters (P_A2520_ and P_A2579_) showing an average Ypet fluorescence increase of greater than 30-fold. The moderate promoters drive expression of genes involved in photosynthesis (A1930, A1961), carbon fixation (A1798), cell wall structure (A2813), and a hypothetical protein (A2531) in *Synechococcus* sp. PCC 7002, while the strong promoters both control hypothetical protein genes (A2520, A2579). Of the two adjacent gene loci analyzed in this study (A1929/A1930 and A1961/A1962), only the most 5′ promoter region showed significant Ypet expression (P_A1930_ and P_A1961_) while expression from the downstream loci promoters (P_A1929_ and P_A1962_) were indistinguishable from the wild type. Both the expression levels and regulatory patterns were inconsistent between the RNA-seq and Ypet expression data. Some of the highest counts per total counts from the RNA-seq data yielded very low Ypet expression (A0740, A1173, A1181, A2062, and A2210) while the second highest Ypet expression level was observed with a promoter from one of the lowest RNA-seq datapoints (A2520). Additionally, many of the engineered strains showed constitutive patterns of Ypet expression, despite the predicted trends from RNA-seq. This lack of correlation between RNA-seq and Ypet expression data is quantified by correlation coefficients, which were calculated across the growth profile (Table 2). Only one gene locus promoter has a correlation coefficient near one (P_A2165_), while many other loci promoters show strong negative correlations.

**Fig. 4:**
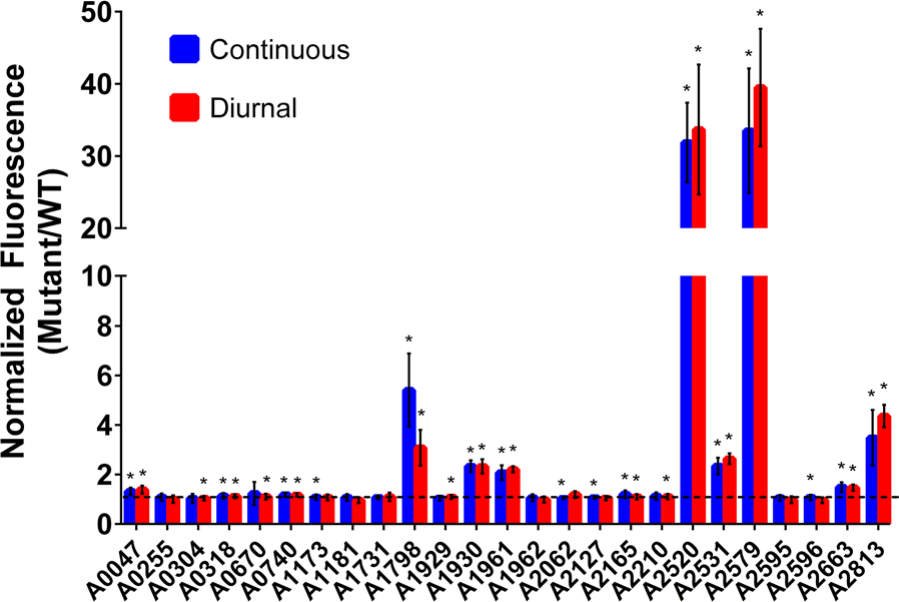
Average normalized expression levels of native *Synechococcus* sp. PCC 7002 promoters, as measured by Ypet fluorescence (Ex 485 nm, Em 528 nm) under continuous and diurnal light conditions. Ypet fluorescence was measured in each engineered strain across a 3 week period of growth and normalized to the wild type fluorescence. The normalized fluorescence values are averaged across the growth period, and at least three biological replicates are included. Normalized Ypet fluorescence values having a change that is statistically significant (two-tail p value <0.05) compared to the wild type are indicated with an *asterisk*. Continuous light conditions are approximately 60 µmol photons m^−2^ s^−1^, and diurnal light conditions consist of 12:12 light dark cycles using the same light intensity

In addition to investigating continuous light conditions, the promoter expression strains were also subjected to diurnal conditions. The only promoter showing a significant change in overall expression levels under diurnal conditions was the *rbc* promoter, with a 43% decrease compared to continuous light conditions (Fig. 4). For all other promoter expression strains, there was no significant change in normalized Ypet fluorescence between continuous light and diurnal conditions. This is not surprising, as the time scale for degradation of the fluorescent protein signal (half-life of 24 h [42]) exceeds the length of the diurnal cycle (12 h light, 12 h dark). Therefore, mRNA levels of *ypet* were measured 6 h after initiation of the light period and 6 h after the start of the dark period for cultures exposed to 5 days of 12:12 diurnal light conditions. The fold-changes in *ypet* expression between light and dark conditions for each promoter expression strain are shown in Table 4. Only two promoters had more than a twofold increase in *ypet* expression under light conditions compared to dark conditions (P_A2520_ and P_A2813_), and two additional promoters had more than a 1.5-fold increase in expression (P_A1731_ and P_A1181_). Of the 25 putative promoters analyzed in this study, only four promoters had reduced expression under light conditions, and the level of decrease in gene expression was less than 35%. The overall average change in gene expression under light conditions for the 25 putative promoters was 1.31 ± 0.34, indicating a slight enhancement of expression in the light.

**Table 4:** Fold-change in *ypet* gene expression under light conditions (6 h after lights on) compared to dark conditions (6 h after lights off) in promoter expression strains, 5 days after inoculation

Strain	Fold-change (light:dark)
A0047	1.01 ± 0.38
A0255	1.01 ± 0.25
A0304	1.29 ± 0.089
A0318	1.39 ± 0.34
A0670	1.31 ± 0.20
A0740	1.44 ± 0.54
A1173	1.41 ± 0.34
A1181	1.79 ± 0.39
A1731	1.85 ± 0.79*
A1798 (*rbc*)	1.16 ± 0.13
A1929	−1.09 ± 0.30
A1930	1.11 ± 0.017
A1961	−1.33 ± 0.30
A1962	1.03 ± 0.39
A2062	1.46 ± 0.23
A2127	1.21 ± 0.40
A2165	1.45 ± 0.015*
A2210	−1.34 ± 0.44
A2520	2.02 ± 0.90
A2531	1.35 ± 0.031
A2579	1.14 ± 0.45
A2595	1.45 ± 0.42
A2596	−1.05 ± 0.10
A2663	1.13 ± 0.31
A2813	2.28 ± 0.73

Gene expression levels were measured by qRT-PCR with 2 biological replicates each. Fold changes with ∆C_T_ values of the light samples having a change that is statistically significant (two-tail p value <0.05) compared to the dark samples are indicated with an asterisk. Positive fold-change values indicate up-regulation under light conditions compared to dark conditions while negative values represent down-regulation under light conditions compared to dark conditions

In an effort to determine the underlying sequences responsible for the expression levels and regulatory patterns observed in this study, we analyzed various groups of the characterized promoter sequences using the online Melina II program [43], which provides analysis from four different motif finder programs (Consensus, MEME, Gibbs, and MDscan). By analyzing all 25 *Synechococcus* sp. PCC 7002 promoters cloned in this study, a common motif was identified by all 4 programs with at least one motif identified in 11 of the putative promoters (Additional file 1: Figure S3A). Analysis of promoter subsets grouped by functional classification (strongly expressed, moderately expressed, and diurnally expressed promoters) revealed only one additional promoter motif in the moderately expressed promoters (Additional file 1: Figure S3B). The functional roles of these putative motifs remain to be investigated experimentally.

## Discussion

The DNA transformation process is the first step in constructing a genetically engineered strain, and as such, this process should be optimized for chassis organisms. In this study, we assessed two tools for improving transformation in *Synechococcus* sp. PCC 7002: neutral integration sites and the homology arm length required for genome integration via homologous recombination. By searching for large gaps in non-coding sequences within the *Synechococcus* sp. PCC 7002 genome, we identified and experimentally confirmed two putative neutral integration sites. It should be noted, however, that only standard growth conditions were tested in this study, and subsequent use of these integration sites under non-standard conditions must be tested with appropriate controls to confirm their neutrality. The longest homology arms tested in this study (1250 bp) yielded the highest transformation efficiency in *Synechococcus* sp. PCC 7002; in practice, however, genetic engineers must balance between improved transformation efficiencies and the expense of longer homology arms—either due to the cost of DNA synthesis or the additional time required to clone larger fragments rather than having them synthesized. The minimum length of homology arms was determined to be 250 bp for *Synechococcus* sp. PCC 7002. Interestingly, this is more than 200 bp longer than the minimum homology arms reported for *E. coli* [44, 45], suggesting that homologous recombination may be less efficient in *Synechococcus* sp. PCC 7002 or that native exonuclease activity may degrade the linearized plasmid containing the integration cassette. This homology arm length is similar to the 300 bp length reported previously for both *Synechocystis* sp. PCC 6803 and *S. elongatus* PCC 7942 [46]. If the minimum homology arm length for genome integration in *Synechococcus* sp. PCC 7002 can be reduced, integration cassette construction may become more efficient, either through a lower cost of synthesizing the DNA cassette or the use of PCR primers to provide the homology arms, as reported previously for *E. coli* [44]. The development of highly efficient DNA transformation and genome integration in *Synechococcus* sp. PCC 7002 will facilitate strain construction in this host and possibly enable the adoption of high throughput genetic engineering techniques [47].

Based on fluorescence excitation and emission properties, three fluorescent protein genes were expressed in *Synechococcus* sp. PCC 7002. We demonstrated the application of these fluorescent proteins as reporters for the quantification of gene expression, as labels for fluorescence microscopy, and as markers for selection via flow cytometry. These techniques may improve our fundamental understanding of *Synechococcus* sp. PCC 7002 and enable the manipulation of this microorganism for biotechnological applications. A potential limitation in the application of fluorescent proteins in cyanobacteria should be considered, however. The native photosynthetic pigments in cyanobacteria, namely the phycobiliproteins and chlorophyll-*a*, may (1) compete with the recombinant fluorescent protein for absorption of the incident light for excitation, (2) re-absorb fluorescence emitted by the fluorescent protein, and (3) emit fluorescence that affects the excitation or emission of the engineered fluorescent protein. This optical interference from native pigments should be carefully considered when designing experiments based on recombinant fluorescent proteins and during the interpretation of results. For example, the relatively low expression of Ypet for most promoter expression strains in this study may be influenced, at least in part, by optical interference from the photosynthetic pigments via reabsorption of the emitted Ypet fluorescence. In our laboratory, we have found a significant reduction in fluorescent protein signals in *Synechococcus* sp. PCC 7002 when compared to common microbial hosts such as *E. coli* (data not shown), yet a full analysis of transcript and protein levels remains to be conducted to confirm the cause of this reduced fluorescence.

In order to develop promoter tools for *Synechococcus* sp. PCC 7002 that can interface with the native regulatory system, we analyzed 24 native promoters selected based on their reported expression level and regulatory pattern from previous RNA-seq data collected by the Bryant laboratory [36]. By measuring fluorescence produced from these promoters in modified *Synechococcus* sp. PCC 7002, we found very poor correlation with the RNA-seq expression data. The discrepancy may be due, in part, to the fact that the fluorescent protein signal has a much longer half-life (>1 day) than the mRNA (<2 min) [48, 49], yet this would only explain an increase in Ypet fluorescence compared to the RNA-seq data. To improve accuracy in expression studies, a degradation tag may be added to the fluorescent protein to reduce the half-life to approximately 1 h, as recently reported in *Synechocystis* sp. PCC 6803 [50]. The lack of correlation between transcript and protein levels is not particularly surprising, given that similar data has been reported for other organisms [51]. Overall, the results of this study suggest that transcriptomic data should not be the basis for promoter tool development in *Synechococcus* sp. PCC 7002.

Characterized promoter tools for recombinant gene expression are essential for chassis development. The *rbc* promoter is commonly used as a strong promoter for expression in cyanobacterial hosts, yet only moderate levels of expression were detected from the *rbc* promoter in *Synechococcus* sp. PCC 7002 (Fig. 4). We identified two strong promoters in *Synechococcus* sp. PCC 7002, P_A2520_ and P_A2579_, which demonstrated more than fivefold higher expression than that measured from the *rbc* promoter. Both of these promoters drive expression of hypothetical protein genes in *Synechococcus* sp. PCC 7002, which may perform important functions based on their high expression levels. While expression level is the typical metric for characterizing promoters, diurnal regulation is also an important metric for a cyanobacterial chassis. By analyzing mRNA levels of *ypet*, P_A2520_ and P_A2813_ were identified as having more than a twofold increase in expression under light conditions compared to dark, while P_A1731_ and P_A1181_ had more moderate, but still significant (>1.5-fold), increases. As such, these promoters may be useful tools for engineering diurnal control of recombinant gene expression. Interestingly, the *rbc* promoter showed reduced Ypet expression under diurnal light conditions (Fig. 4), yet there was no significant change in the ratio of *ypet* expression under light vs. dark conditions (Table 4). This suggests that expression from the *rbc* promoter may be constitutively lower under diurnal conditions or that a finer time resolution of transcript levels is needed to detect diurnal changes. Overall, this study identified and characterized two strong promoters and five moderate promoters as tools for controlling gene expression in *Synechococcus* sp. PCC 7002. Furthermore, two strong and two moderate promoters were determined to have diurnally regulated expression, with increased expression under light conditions.

Genetic tool development for cyanobacterial chassis is still at an early stage. This study, along with other recent efforts in cyanobacterial tool development [26, 28], aims to advance *Synechococcus* sp. PCC 7002 as a ‘green’ chassis, enabling the biological production of useful metabolites from CO_2_ and sunlight to support industries including biofuels, nutraceuticals, and specialty chemicals. In addition to industrial applications, these genetic tools will help to advance our fundamental understanding of cyanobacteria and their functional role in the Earth’s ecosystems.

## Additional file

**Additional file 1.** Additional information.
