# Supplementary material for: Genetic tools for advancement of Synechococcus sp. PCC 7002 as a cyanobacterial chassis
Source: Microb Cell Fact. 2016 Nov 10;15:190. doi: 10.1186/s12934-016-0584-6 (PMC5105302; doi:10.1186/s12934-016-0584-6)
Supplement: Supplementary file 1 — Additional file 1. Additional information. [file 12934_2016_584_MOESM1_ESM.docx]

Additional Information

Genetic Tools for Advancement of *Synechococcus* sp. PCC 7002 as a Cyanobacterial Chassis

Contents: Supplemental [Figures](#_Figures), [Tables](#_Tables), and [DNA Sequences](#_DNA_Sequences).

# Figures





**Figure S1. Fluorescence difference spectra (mutant – wild type) to determine the emission (dashed lines) and excitation (solid lines) maxima for mutant *Synechococcus* sp. PCC 7002 expressing hGFP, Ypet, and mOrange. The wild type and mutants were excited at 465 nm (hGFP), 485 nm (Ypet), and 515 nm (mOrange), and excitation spectra were collected for emission wavelengths of 520 nm (hGFP), 565 nm (Ypet), and 600 nm (mOrange).**

**
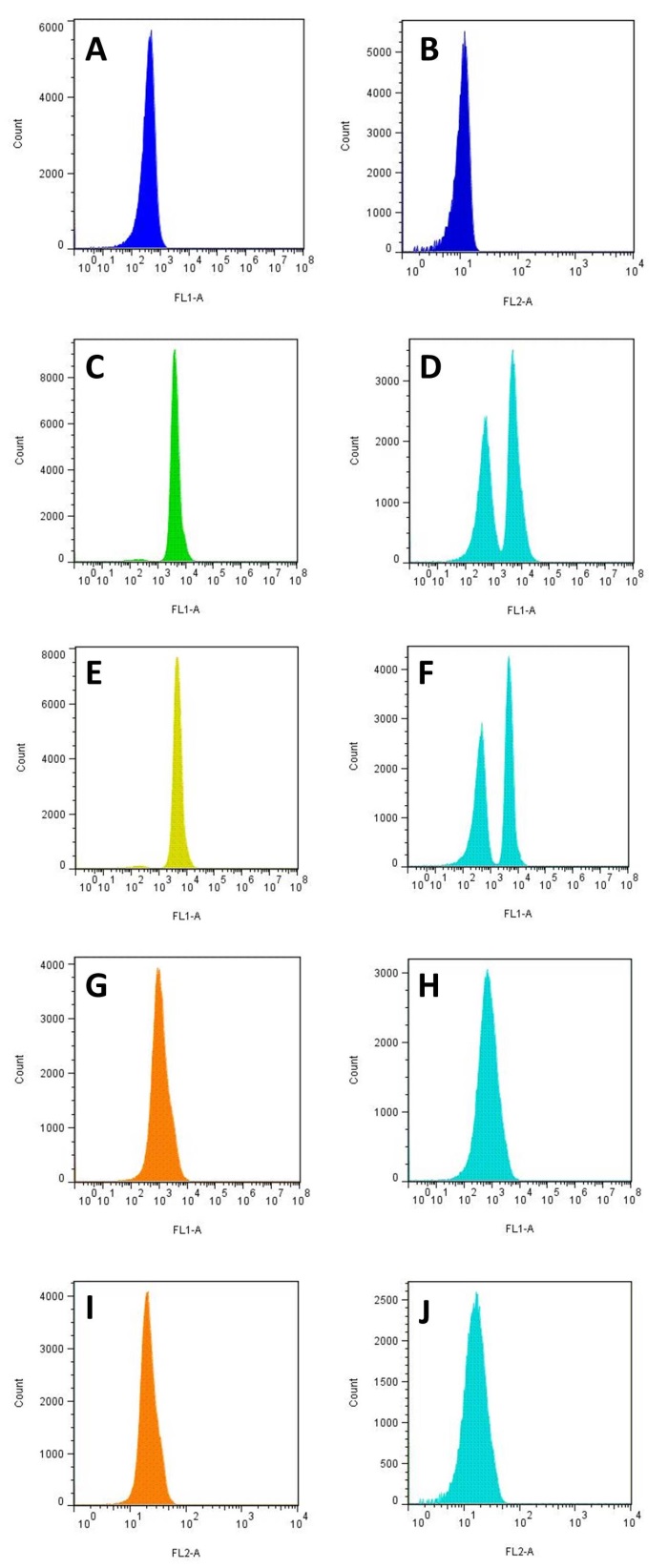
**

**Figure S2. Flow cytometer counts for wild type (A), 7002-hGFP (C), 7002-Ypet (E), and 7002-mOrange (G) at 533 nm, and a 50/50 mixture of wild type and wild type (B), 7002-hGFP (D), 7002-Ypet (F), and 7002-mOrange (H) at 533 nm. For mOrange, counts were also measured at 585 nm for 7002-mOrange (I) and a 50/50 mixture of wild type and 7002-mOrange (J).**

**
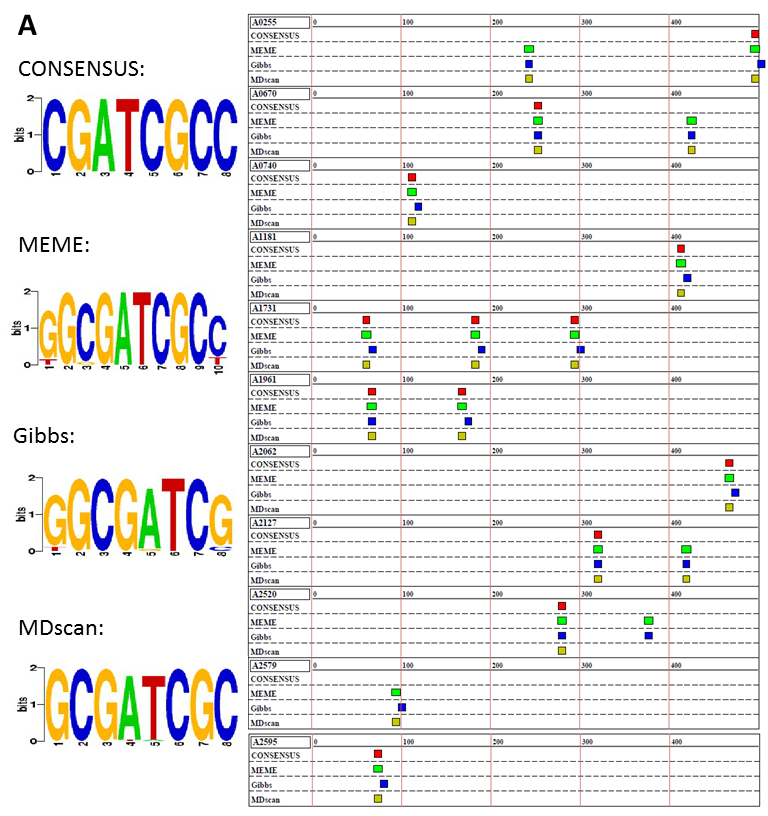

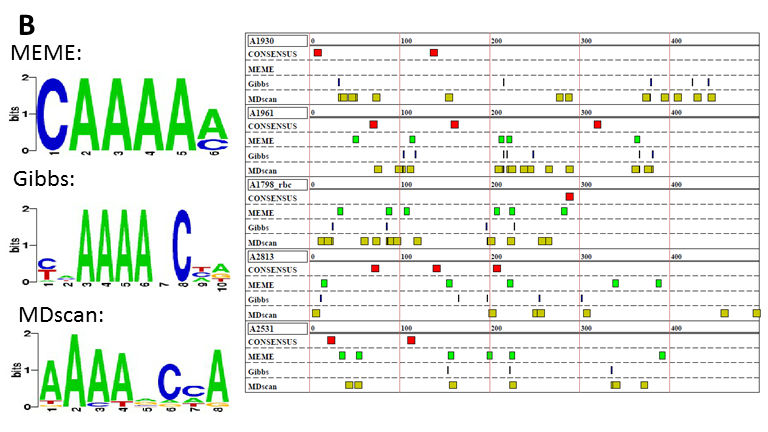
**

**Figure S3. Promoter motif analysis using Melina II. Motif sequence and locations for a motif common in 11 of the 25 promoters (A) and a motif identified in moderately expressed promoters (B).**

# Tables

**Table S1. Primers used in this study**

| **Primer Name** | **Sequence** | **Description** |
| --- | --- | --- |
| NS1_5F | CAAGCCCAAGGGTTTCGTAGGGATT | To amplify the 5’ homology fragment for NS1 |
| NS1_5R | ACGGTTTACAAGCATAAAGCTCTAGAGATCGAAAGAAAGAGGATCCATAA |  |
| NS1_3F | aacggtttacaagcataaagctctagttatagaaaaaagcttcattatatagaa | To amplify the 3’ homology fragment for NS1 |
| NS1_3R | TTGCGAATGTGATTGTAGAAGAATTGAATTTAGC |  |
| SpR_F | ctagagctttatgcttgtaaaccgt | To amplify the spectinomycin resistance cassette from pAM2991 |
| SpR_R | TTTTTAAAATAAAAAAGGGGACCTC |  |
| NS2_5F | TGGGGGCTTGTTGAAGATTTACTTTAGGCA | To amplify the 5’ homology fragment for NS2 |
| NS2_5R | GCTGGCAATTCCGACGTCTAAGTCTTAATTTATGGGCATCTCCCTG |  |
| NS2_3F | GCGGGACTCTGGGGTTCGAccttgtgatgatcactaccgtttaa | To amplify the 3’ homology fragment for NS2 |
| NS2_3R | TTTGAGGTAATTTTCGTTAATTCTAACCATAGAA |  |
| KmR_F | TTAGACGTCGGAATTGCCAGC | To amplify the kanamycin resistance cassette from pSB |
| KmR_R | TCGAACCCCAGAGTCCCGC |  |
| desB1250_5F | ACTGATGAGCTCTGGGTCTGTCTCTGGCTTC | To amplify a 1250 bp 5’ homology arm for integration at *desB* |
| desB750_5F | CTGACTGAGCTCTCCCTAATACTTCACCATGAT | To amplify a 750 bp 5’ homology arm for integration at *desB* |
| desB500_5F | TACTAGGAGCTCAAGCCAAAAAAGCGAATTA | To amplify a 500 bp 5’ homology arm for integration at *desB* |
| desB250_5F | CTAGCTGAGCTCCGAGGGTAATAGGTAAGGTAA | To amplify a 250 bp 5’ homology arm for integration at *desB* |
| desB5R | TGTGACGAGCTCTCAGTTTCAAAAAGAGATTAACA | To amplify 1250, 750, 500, and 250 bp 5’ homology arms for integration at *desB* |
| desB3F | TCAGTACCTAGGGCTCCAAAAGCGTGACTAGAT | To amplify 1250, 750, 500, and 250 bp 3’ homology arms for integration at *desB* |
| desB1250_3R | TCTTGTCCTAGGTCAGCTTGTTGCCAGCGGTA | To amplify a 1250 bp 3’ homology arm for integration at *desB* |
| desB750_3R | CTAGTACCTAGGATATTTGAGGCGTAGCTCCAAC | To amplify a 750 bp 3’ homology arm for integration at *desB* |
| desB500_3R | CATGTTCCTAGGTTAATCAAAATATGAGGCGTTAACA | To amplify a 500 bp 3’ homology arm for integration at *desB* |
| desB250_3R | GTACAACCTAGGCTGGAAACAAGGGAAGTAAAG | To amplify a 250 bp 3’ homology arm for integration at *desB* |
| TrbcR | GCTGGCAATTCCGACGTCTAAAATCACTACCATGGCGTTGA | Used with NS2_5F to amplify fluorescent protein gBlocks fragment for overlap PCR |
| NS2_5reF | GAACATGAGCTCtgggggcttgttgaagatttactttaggca | To amplify NS2_5’-P_rbc_-Ypet-T_rbc_-KmR-NS2_3’ for ligation with the origin of replication from pSB |
| NS2_3reR | CATCGTCCTAGGTTTGAGGTAATTTTCGTTAATTCTAACCATAGAA |  |
| pYpetF | P-CTGTTGCATATGATGAGTAAAGGCGAAGAACTCT | To remove Prbc from pSBP_rbc_Ypet and insert KpnI and NdeI restriction sites upstream of Ypet. |
| pYpetR | P-GTACATGGTACCGTCTTAATTTATGGGCATCTC |  |
| A0047F | CTGGATGGTACCTACTTAAGGGTTGCTCTCAAACAAGA | To amplify the 500 bp upstream of Synpcc7002_A0047 |
| A0047R | CGGATACATATGAAACAACGCTCCAAATCAAACTTC |  |
| A0255F | CTGTGACATATGGGCGATCGTCCGCAAATA | To amplify the 500 bp upstream of Synpcc7002_A0255 |
| A0255R | CTGTGACATATGGGCGATCGCCCGCAAATA |  |
| A0304F | AAGTCTGGTACCcgatcgccctccttgtagt | To amplify the 500 bp upstream of Synpcc7002_A0304 |
| A0304R | GACTGACATATGAAAGTAAGGCTTAAACCTCCTTGTTG |  |
| A0318F | TGCAAAGGTACCagatcgtcaccgccgagca | To amplify the 500 bp upstream of Synpcc7002_A0318 |
| A0318R | AGTCAACATATGGTCCAAACACCAGATCAAACGAA |  |
| A0670F | GCTCTAGGTACCatattcacattcggccaatctgcga | To amplify the 500 bp upstream of Synpcc7002_A0670 |
| A0670R | GTACTACATATGATAAAAAGGCGGTGATGGGCTTAAATC |  |
| A0740F | CAGTATGGTACCtaacatcagtttgaatggagtgaaa | To amplify the 500 bp upstream of Synpcc7002_A0740 |
| A0740R | TGAACTCATATGTGGGGTGTTACTTGCCGAA |  |
| A1173F | TACTGAGGTACCggtggagctgatcctgaccc | To amplify the 500 bp upstream of Synpcc7002_A1173 |
| A1173R | CGTGGTCATATGGTATGCCTTAGCAACTCCTGTGAA |  |
| A1181F | TATCACGGTACCCGGTGGGCTGGGTACAGTAG | To amplify the 500 bp upstream of Synpcc7002_A1181 |
| A1181R | TGGCAGCATATGGGGTTTAGTCCTGACGTTCTAAAA |  |
| A1731F | ACCTATGGTACCtagcgaaagttggcaaaatacg | To amplify the 500 bp upstream of Synpcc7002_A1731 |
| A1731R | TCGTAGCATATGGGGGATTTATCTTTTGTGAGCG |  |
| A1929F | ATTCAAGGTACCgatcgcatcaaagcttttgttac | To amplify the 500 bp upstream of Synpcc7002_A1929 |
| A1929R | GGCGGTCATATGTGTTATAATTCCTTATCTTAGTAGGTCTTAA |  |
| A1930F | AATATTGGTACCtcacccccgaaccagaac | To amplify the 500 bp upstream of Synpcc7002_A1930 |
| A1930R | GATCAACATATGCTATGGATTCCTCCTCTAGATATGAG |  |
| A1961F | CAATGAGGTACCcgcatttgaggggagaggtc | To amplify the 500 bp upstream of Synpcc7002_A1961 |
| A1961R | TGGACTCATATGGAGGATTCTCCTCTCTAGACAATCG |  |
| A1962F | TAGACTGGTACCGCTGGCTCCGTGACTTCCTC | To amplify the 500 bp upstream of Synpcc7002_A1962 |
| A1962R | GGACTCCATATGAACTATGCTTGAGGTTCTCCTCGT |  |
| A2062F | ACTGAAGGTACCaaacagcaatccgtaacctgacTC | To amplify the 500 bp upstream of Synpcc7002_A2062 |
| A2062R | GGACCACATATGGATGACCTCCTGCAAACTGATTTT |  |
| A2127F | CTTATCGGTACCagctcagtcatccctgggaaat | To amplify the 500 bp upstream of Synpcc7002_A2127 |
| A2127R | GACTATCATATGGGTGTTCCGTTGGCGTTTG |  |
| A2165F | TAACATGGTACCCGCGCCCTCCACTTTTTC | To amplify the 500 bp upstream of Synpcc7002_A2165 |
| A2165R | GAGCCTCATATGTGTATTTTTCCCATTTTTTATTCAATAGTT |  |
| A2210F | ATATAAGGTACCgccgcatgaccaacaatg | To amplify the 500 bp upstream of Synpcc7002_A2210 |
| A2210R | CGTCAGCATATGTGTCAGATTATCTCCCTATGGATTA |  |
| A2520F | TTACTAGGTACCggaaaacccgatggtgcc | To amplify the 500 bp upstream of Synpcc7002_A2520 |
| A2520R | GCACTGCATATGATTTTAATTTTTGTTAACTTGGCTCCAT |  |
| A2531F | TTCACTGGTACCgccacacggcaaaggtaatc | To amplify the 500 bp upstream of Synpcc7002_A2531 |
| A2531R | GACCTCCATATGCTTTTTATCCTCACACATTAAATTTTAG |  |
| A2579F | ATTAATGGTACCgggtaggccaaaaattgcc | To amplify the 500 bp upstream of Synpcc7002_A2579 |
| A2579R | GCACGTCATATGTTTTGTTTCTCCAATAATCAAAATT |  |
| A2595F | TGAACTGGTACCTAGATGTCACTGAGCTTTCTGAAAC | To amplify the 500 bp upstream of Synpcc7002_A2595 |
| A2595R | GCTTACCATATGTACCTGAAGTGTGATCCGATGA |  |
| A2596F | GATTACGGTACCTCAGCCCCAGTTGCGTCC | To amplify the 500 bp upstream of Synpcc7002_A2596 |
| A2596R | GTCTACCATATGAGGACAAGGAAGAAAAGAAGTAATGG |  |
| A2663F | ATCTTAGGTACCaataaatccaaaaccttctctaaaaca | To amplify the 500 bp upstream of Synpcc7002_A2663 |
| A2663R | GTAGCTCATATGTTCGCCTTATTTCACTCCAAAT |  |
| A2813F | TCTGTAGGTACCCCGATTTAAGTTCAAAAACTTTATTG | To amplify the 500 bp upstream of Synpcc7002_A2813 |
| A2813R | CGTCACCATATGTTGTTTTTCCTCACACATTAAGGC |  |
| qYpetF | CCGAAGGCTATGTGCAAGAA | To amplify *ypet* fragment for qRT-PCR |
| qYpetR | CGAGTTTGTGGCCGAGAATA |  |
| qrnpAF | GCAATCATTCTCTTACCTCAGCC | To amplify *rnpA* fragment for qRT-PCR |
| qrnpAR | TACCGCACAGCAATGACGAT |  |
| desBscF | CTGGTTTGCGGTTTCTAATTG | To screen for integration of Km^R^ cassette at *desB* |
| desBscR | gctgaccgcttcctcgtg |  |

**Table S2. Plasmids used and constructed in this study.**

| **Plasmid** | **Description** | **Reference** |
| --- | --- | --- |
| pSB | Plasmid for genome integration at *desB* in *Synechococcus* sp. PCC 7002 with homology arms of 1000 bp | (Ruffing, 2014) |
| pSB1250 | Plasmid for genome integration at *desB* in *Synechococcus* sp. PCC 7002 with homology arms of 1250 bp | This study |
| pSB750 | Plasmid for genome integration at *desB* in *Synechococcus* sp. PCC 7002 with homology arms of 750 bp | This study |
| pSB500 | Plasmid for genome integration at *desB* in *Synechococcus* sp. PCC 7002 with homology arms of 500 bp | This study |
| pSB250 | Plasmid for genome integration at *desB* in *Synechococcus* sp. PCC 7002 with homology arms of 250 bp | This study |
| pSBP_rbc_Ypet | Modified pSB to include codon optimized Ypet with *rbc* promoter and terminator | This study |
| pSBYpet | Modified pSBP_rbc_Ypet with removal of P_rbc_ and insertion of KpnI and NdeI upstream of Ypet for promoter insertion | This study |
| pSBP_0047_Ypet | Modified pSBYpet with insertion of 500 bp upstream of Synpcc7002_A0047 to drive Ypet expression | This study |
| pSBP_0255_Ypet | Modified pSBYpet with insertion of 500 bp upstream of Synpcc7002_A0255 to drive Ypet expression | This study |
| pSBP_0304_Ypet | Modified pSBYpet with insertion of 500 bp upstream of Synpcc7002_A0304 to drive Ypet expression | This study |
| pSBP_0318_Ypet | Modified pSBYpet with insertion of 500 bp upstream of Synpcc7002_A0318 to drive Ypet expression | This study |
| pSBP_0670_Ypet | Modified pSBYpet with insertion of 500 bp upstream of Synpcc7002_A0670 to drive Ypet expression | This study |
| pSBP_0740_Ypet | Modified pSBYpet with insertion of 500 bp upstream of Synpcc7002_A0740 to drive Ypet expression | This study |
| pSBP_1173_Ypet | Modified pSBYpet with insertion of 500 bp upstream of Synpcc7002_A1173 to drive Ypet expression | This study |
| pSBP_1181_Ypet | Modified pSBYpet with insertion of 500 bp upstream of Synpcc7002_A1181 to drive Ypet expression | This study |
| pSBP_1731_Ypet | Modified pSBYpet with insertion of 500 bp upstream of Synpcc7002_A1731 to drive Ypet expression | This study |
| pSBP_1929_Ypet | Modified pSBYpet with insertion of 500 bp upstream of Synpcc7002_A1929 to drive Ypet expression | This study |
| pSBP_1930_Ypet | Modified pSBYpet with insertion of 500 bp upstream of Synpcc7002_A1930 to drive Ypet expression | This study |
| pSBP_1961_Ypet | Modified pSBYpet with insertion of 500 bp upstream of Synpcc7002_A1961 to drive Ypet expression | This study |
| pSBP_1962_Ypet | Modified pSBYpet with insertion of 500 bp upstream of Synpcc7002_A1962 to drive Ypet expression | This study |
| pSBP_2062_Ypet | Modified pSBYpet with insertion of 500 bp upstream of Synpcc7002_A2062 to drive Ypet expression | This study |
| pSBP_2127_Ypet | Modified pSBYpet with insertion of 500 bp upstream of Synpcc7002_A2127 to drive Ypet expression | This study |
| pSBP_2165_Ypet | Modified pSBYpet with insertion of 500 bp upstream of Synpcc7002_A2165 to drive Ypet expression | This study |
| pSBP_2210_Ypet | Modified pSBYpet with insertion of 500 bp upstream of Synpcc7002_A2210 to drive Ypet expression | This study |
| pSBP_2520_Ypet | Modified pSBYpet with insertion of 500 bp upstream of Synpcc7002_A2520 to drive Ypet expression | This study |
| pSBP_2531_Ypet | Modified pSBYpet with insertion of 500 bp upstream of Synpcc7002_A2531 to drive Ypet expression | This study |
| pSBP_2579_Ypet | Modified pSBYpet with insertion of 500 bp upstream of Synpcc7002_A2579 to drive Ypet expression | This study |
| pSBP_2595_Ypet | Modified pSBYpet with insertion of 500 bp upstream of Synpcc7002_A2595 to drive Ypet expression | This study |
| pSBP_2596_Ypet | Modified pSBYpet with insertion of 500 bp upstream of Synpcc7002_A2596 to drive Ypet expression | This study |
| pSBP_2663_Ypet | Modified pSBYpet with insertion of 500 bp upstream of Synpcc7002_A2663 to drive Ypet expression | This study |
| pSBP_2813_Ypet | Modified pSBYpet with insertion of 500 bp upstream of Synpcc7002_A2813 to drive Ypet expression | This study |

# DNA Sequences

NS1_KO fragment (NS1 homology regions in bold and SpR cassette with start and stop codons underlined):

**CAAGCCCAAGGGTTTCGTAGGGATTTTGTTCGCTCATtctcctcctcctgtgagcatgccactgggttattggaacatccttagattcttaatgtaacccaatcatcatacagtttattattatgagcgttttccggtgcgctttccatagagcaaaattttttctggtgatgatagccggggcgatcgcctcaagacttgcccagaccagagagtatacatccccatacttaacgctagctgatcaacggagtaggtactcgaaccactcaggctccctaaaccgctgcaatgtacaaaatcttagagaccatcccaaagaccctccactaagcgatcgtgtagaggccactgaacagacaagcacaaaaatacaatcctcccagactatggtttgtgtccgttgatcgcactcggcatgaaaataagtccattttgtcgattgaccctaccataaaaactcaagcttgatcttTTATGGATCCTCTTTCTTTCGATCT**CTAGAGCTTTATGCTTGTAAACCGTtttgtgaaaaaatttttaaaataaaaaaggggacctctagggtccccaattaattagtaatataatctattaaaggtcattcaaaaggtcatccaccggatcaattcccctgctcgcgcaggctgggtgccaagctctcgggtaacatcaaggcccgatccttggagcccttgccctcccgcacgatgatcgtgccgtgatcgaaatccagatccttgacccgcagttgcaaaccctcactgatccgcatgcccgttccatacagaagctgggcgaacaaacgatgctcgccttccagaaaaccgaggatgcgaaccacttcatccggggtcagcaccaccggcaagcgccgcgacggccgaggtcttccgatctcctgaagccagggcagatccgtgcacagcaccttgccgtagaagaacagcaaggccgccaatgcctgacgatgcgtggagaccgaaaccttgcgctcgttcgccagccaggacagaaatgcctcgacttcgctgctgcccaaggttgccgggtgacgcacaccgtggaaacggatgaaggcacgaacccagtggacataagcctgttcggttcgtaagctgtaatgcaagtagcgtatgcgctcacgcaactggtccagaaccttgaccgaacgcagcggtggtaacggcgcagtggcggttttcatggcttgttatgactgtttttttggggtacagtctatgcctcgggcatccaagcagcaagcgcgttacgccgtgggtcgatgtttgatgttatggagcagcaacgatgttacgcagcagggcagtcgccctaaaacaaagttaaacatcatgagggaagcggtgatcgccgaagtatcgactcaactatcagaggtagttggcgtcatcgagcgccatctcgaaccgacgttgctggccgtacatttgtacggctccgcagtggatggcggcctgaagccacacagtgatattgatttgctggttacggtgaccgtaaggcttgatgaaacaacgcggcgagctttgatcaacgaccttttggaaacttcggcttcccctggagagagcgagattctccgcgctgtagaagtcaccattgttgtgcacgacgacatcattccgtggcgttatccagctaagcgcgaactgcaatttggagaatggcagcgcaatgacattcttgcaggtatcttcgagccagccacgatcgacattgatctggctatcttgctgacaaaagcaagagaacatagcgttgccttggtaggtccagcggcggaggaactctttgatccggttcctgaacaggatctatttgaggcgctaaatgaaaccttaacgctatggaactcgccgcccgactgggctggcgatgagcgaaatgtagtgcttacgttgtcccgcatttggtacagcgcagtaaccggcaaaatcgcgccgaaggatgtcgctgccgactgggcaatggagcgcctgccggcccagtatcagcccgtcatacttgaagctagacaggcttatcttggacaagaagaagatcgcttggcctcgcgcgcagatcagttggaagaatttgtccactacgtgaaaggcgagatcaccaaggtagtcggcaaataatgtctaacaattcgttcaagccgacgccgcttcgcggcgcggcttaactcaagcgttagatgcactaagcacataattgctcacagccaaactatcaggtcaagtctgcttttattatttttaagcgtgcataataagccctacacaaattgggagatatatcatgaaaggctggctttttcttgttatcgcaatagttggcgaagtaatcgcaacatccgcattaaaatctagcgagggctttactaagctgatccggtggatgaccttttgaatgacctttaatagattatattactaattaattggggaccctagaggtccccttttttattttaaaaattttttcacaaAACGGTTTACAAGCATAAAGCTCTAG**ttatagaaaaaagcttcattatatagaatttctaaaaatcaagctttaaagggttgaccttccttttgaaaggtaataaaactgaagtacaaacaaatacgtaagtaacgaaaaggagtaagccgcaatctgcctcagtagaaacacgtatttttaccccaaaagaagtattctattcatctttctcctttgtgagaaggttcatggagatccttctggggcacttgaaacctctacttgcaaggatcaaagcgacatatgtacgtgtttttccaaaaaaatcccagaagtagggagtaattcctctactgagatttcaagaaaattaagtgttctgaggcttggtttatacgggaaatttagacttgatggtttgggtcttaccttttcctagcagcaagactaaattaattagttacgcatttgttgaaatcaaactgaattctattgaggattccttacaATGGCTAAATTCAATTCTTCTACAATCACATTCGCAA**

NS2_KO fragment (NS2 homology regions in bold and KmR cassette with start and stop codons underlined):

**tGGGGGCTTGTTGAAGATTTACTTTAGGCATTtacgctttaactccttaaatctaaagaacgaagaactatatggtgcggcccaggggggcagatactttacaaaacaatcatcatgtaggtcacaatctagaacacaaagaatttttctaaaaaataaacgacataccctttaactttcttacgctttataacttttaattgagcgctcactactttagacgggcaacaatcgaaaaaggttcaacgattcattaaaatctcagggatggggaattcatcggcgttctatacttaattctccttgattgaccaagagcttgaaaaggggaaaagatgcgggattaccgaacttggaatatcgacagactcaattagaaatatagtgtcgttgcaatatgttattcaatgcgataagggtggcccttgcggggaagacatctctcaaaaatgagacctctattgcagtagaggtaCAGGGAGATGCCCATAAATTAAGAC**TTAGACGTCGGAATTGCCAGCTGGGGCGCCCTCTGGTAAGGTTGGGAAGCCCTGCAAAGTAAACTGGATGGCTTTCTTGCCGCCAAGGATCTGATGGCGCAGGGGATCAAGATCTGATCAAGAGACAGGATGAGGATCGTTTCGCATGATTGAACAAGATGGATTGCACGCAGGTTCTCCGGCCGCTTGGGTGGAGAGGCTATTCGGCTATGACTGGGCACAACAGACAATCGGCTGCTCTGATGCCGCCGTGTTCCGGCTGTCAGCGCAGGGGCGCCCGGTTCTTTTTGTCAAGACCGACCTGTCCGGTGCCCTGAATGAACTGCAGGACGAGGCAGCGCGGCTATCGTGGCTGGCCACGACGGGCGTTCCTTGCGCAGCTGTGCTCGACGTTGTCACTGAAGCGGGAAGGGACTGGCTGCTATTGGGCGAAGTGCCGGGGCAGGATCTCCTGTCATCTCACCTTGCTCCTGCCGAGAAAGTATCCATCATGGCTGATGCAATGCGGCGGCTGCATACGCTTGATCCGGCTACCTGCCCATTCGACCACCAAGCGAAACATCGCATCGAGCGAGCACGTACTCGGATGGAAGCCGGTCTTGTCGATCAGGATGATCTGGACGAAGAGCATCAGGGGCTCGCGCCAGCCGAACTGTTCGCCAGGCTCAAGGCGCGCATGCCCGACGGCGAGGATCTCGTCGTGACCCATGGCGATGCCTGCTTGCCGAATATCATGGTGGAAAATGGCCGCTTTTCTGGATTCATCGACTGTGGCCGGCTGGGTGTGGCGGACCGCTATCAGGACATAGCGTTGGCTACCCGTGATATTGCTGAAGAGCTTGGCGGCGAATGGGCTGACCGCTTCCTCGTGCTTTACGGTATCGCCGCTCCCGATTCGCAGCGCATCGCCTTCTATCGCCTTCTTGACGAGTTCTTCTGAGCGGGACTCTGGGGTTCGA**ccttgtgatgatcactaccgtttaaaagattgaaaaagaatatttttttgaccaataagtccagtttattcaaaaaaaggcgctttgtatacctttaggaaaaagataatctcagcaaaaataagggcatttaggccagaaatgaagaacgtagaagtattttggcaagccagggatttcacctaaaaattaacggttgataatccttctcaaaaataaaagaatgtccttgttagtccaggaaaccgaggtggcgatcgcctttggatcaagatctattcccagagttgcagaatttgtgccgtgatgggatggcgtttcgatttttgtaacccggtgcgatggattggtcaggtgagtagcgatggataatttttccggcctcttcaccccaaaaagatctagcggcttccctggggcatgggatgataggttaagctgtcttacggtcttttgcccctgatttTTCTATGGTTAGAATTAACGAAAATTACCTCAAA**

NS2_5’-P_rbc_-hGFP-T_rbc_-KmR-NS2_3’ (NS2 homology regions in bold, hGFP underlined, and KmR cassette in italics)

**TGGGGGCTTGTTGAAGATTTACTTTAGGCATTTACGCTTTAACTCCTTAAATCTAAAGAA**

**CGAAGAACTATATGGTGCGGCCCAGGGGGGCAGATACTTTACAAAACAATCATCATGTAG**

**GTCACAATCTAGAACACAAAGAATTTTTCTAAAAAATAAACGACATACCCTTTAACTTTC**

**TTACGCTTTATAACTTTTAATTGAGCGCTCACTACTTTAGACGGGCAACAATCGAAAAAG**

**GTTCAACGATTCATTAAAATCTCAGGGATGGGGAATTCATCGGCGTTCTATACTTAATTC**

**TCCTTGATTGACCAAGAGCTTGAAAAGGGGAAAAGATGCGGGATTACCGAACTTGGAATA**

**TCGACAGACTCAATTAGAAATATAGTGTCGTTGCAATATGTTATTCAATGCGATAAGGGT**

**GGCCCTTGCGGGGAAGACATCTCTCAAAAATGAGACCTCTATTGCAGTAGAGGTACAGGG**

**AGATGCCCATAAATTAAGAC**TCGAGCGGGATTTTATGGCTTTTTTAGGTATTTTTGTAAG

GGTAAAATAGGCCCATCAAACAGCATTAGAAATGCTAATCAGCCCAAAAAACAAAAGCAA

TCTTTTTTTGTTGCTAAAAGATAAAAATAAGTCGAGGCTGTGGTAACATATCCCACAGAT

TAAAGAAAGTCATAAGACTTGAATCTTCAGAATTTTAAAAAGCAGTTTTGCCAACGTAAG

ATTTTTGAAGTTTTCGACCAACAATACCGTTACTGGTATTTGTCTGTTAAAGATAAGCAT

TTTTGCTGGAGGAAAACCGCATGGTGAGTAAAGGCGAAGAACTCTTTACCGGCGTGGTGC

CCATTCTCGTGGAACTCGATGGCGATGTGAATGGCCACAAATTTAGTGTGAGTGGCGAAG

GCGAAGGCGATGCCACCTATGGCAAACTCACCCTCAAATTTATTTGTACCACCGGCAAAC

TCCCCGTGCCCTGGCCCACCCTCGTGACCACCTTTGCCTATGGCCTCCAATGTTTTGCCC

GCTATCCCGATCACATGAAACAACACGATTTTTTTAAAAGTGCCATGCCCGAAGGCTATG

TGCAAGAACGCACCATTTTTTTTAAAGATGATGGCAATTATAAAACCCGCGCCGAAGTGA

AATTTGAAGGCGATACCCTCGTGAATCGCATTGAACTCAAAGGCATTGATTTTAAAGAAG

ATGGCAATATTCTCGGCCACAAACTCGAATATAATTATAATAGTCACAATGTGTATATTA

TGGCCGATAAACAAAAAAATGGCATTAAAGTGAATTTTAAAATTCGCCACAATATTGAAG

ATGGCAGTGTGCAACTCGCCGATCACTATCAACAAAATACCCCCATTGGCGATGGCCCCG

TGCTCCTCCCCGATAATCACTATCTCAGTACCCAAAGTGCCCTCAGTAAAGATCCCAATG

AAAAACGCGATCACATGGTGCTCCTCGAATTTGTGACCGCCGCCGGCATTACCCTCGGCA

TGGATGAACTCTATAAATTATAATTTGAATCATGCGGAATGCGATCGCCTTAGGACGGTC

GCATTTTTTGTTTACGTCTAAAATTAGTCGAAATCCCCCATCAACGCCATGGTAGTGATT *TTAGACGTCGGAATTGCCAGCTGGGGCGCCCTCTGGTAAGGTTGGGAAGCCCTGCAAAGT*

*AAACTGGATGGCTTTCTTGCCGCCAAGGATCTGATGGCGCAGGGGATCAAGATCTGATCA*

*AGAGACAGGATGAGGATCGTTTCGCATGATTGAACAAGATGGATTGCACGCAGGTTCTCC*

*GGCCGCTTGGGTGGAGAGGCTATTCGGCTATGACTGGGCACAACAGACAATCGGCTGCTC*

*TGATGCCGCCGTGTTCCGGCTGTCAGCGCAGGGGCGCCCGGTTCTTTTTGTCAAGACCGA*

*CCTGTCCGGTGCCCTGAATGAACTGCAGGACGAGGCAGCGCGGCTATCGTGGCTGGCCAC*

*GACGGGCGTTCCTTGCGCAGCTGTGCTCGACGTTGTCACTGAAGCGGGAAGGGACTGGCT*

*GCTATTGGGCGAAGTGCCGGGGCAGGATCTCCTGTCATCTCACCTTGCTCCTGCCGAGAA*

*AGTATCCATCATGGCTGATGCAATGCGGCGGCTGCATACGCTTGATCCGGCTACCTGCCC*

*ATTCGACCACCAAGCGAAACATCGCATCGAGCGAGCACGTACTCGGATGGAAGCCGGTCT*

*TGTCGATCAGGATGATCTGGACGAAGAGCATCAGGGGCTCGCGCCAGCCGAACTGTTCGC*

*CAGGCTCAAGGCGCGCATGCCCGACGGCGAGGATCTCGTCGTGACCCATGGCGATGCCTG*

*CTTGCCGAATATCATGGTGGAAAATGGCCGCTTTTCTGGATTCATCGACTGTGGCCGGCT*

*GGGTGTGGCGGACCGCTATCAGGACATAGCGTTGGCTACCCGTGATATTGCTGAAGAGCT*

*TGGCGGCGAATGGGCTGACCGCTTCCTCGTGCTTTACGGTATCGCCGCTCCCGATTCGCA*

*GCGCATCGCCTTCTATCGCCTTCTTGACGAGTTCTTCTGAGCGGGACTCTGGGGTTCGA***c**

**cttgtgatgatcactaccgtttaaaagattgaaaaagaatatttttttgaccaataagtc**

**cagtttattcaaaaaaaggcgctttgtatacctttaggaaaaagataatctcagcaaaaa**

**taagggcatttaggccagaaatgaagaacgtagaagtattttggcaagccagggatttca**

**cctaaaaattaacggttgataatccttctcaaaaataaaagaatgtccttgttagtccag**

**gaaaccgaggtggcgatcgcctttggatcaagatctattcccagagttgcagaatttgtg**

**ccgtgatgggatggcgtttcgatttttgtaacccggtgcgatggattggtcaggtgagta**

**gcgatggataatttttccggcctcttcaccccaaaaagatctagcggcttccctggggca**

**tgggatgataggttaagctgtcttacggtcttttgcccctgatttTTCTATGGTTAGAAT**

**TAACGAAAATTACCTCAAA**

NS2_5’-P_rbc_-Ypet-T_rbc_-KmR-NS2_3’ (NS2 homology regions in bold, Ypet underlined, and KmR cassette in italics)

**TGGGGGCTTGTTGAAGATTTACTTTAGGCATTTACGCTTTAACTCCTTAAATCTAAAGAA**

**CGAAGAACTATATGGTGCGGCCCAGGGGGGCAGATACTTTACAAAACAATCATCATGTAG**

**GTCACAATCTAGAACACAAAGAATTTTTCTAAAAAATAAACGACATACCCTTTAACTTTC**

**TTACGCTTTATAACTTTTAATTGAGCGCTCACTACTTTAGACGGGCAACAATCGAAAAAG**

**GTTCAACGATTCATTAAAATCTCAGGGATGGGGAATTCATCGGCGTTCTATACTTAATTC**

**TCCTTGATTGACCAAGAGCTTGAAAAGGGGAAAAGATGCGGGATTACCGAACTTGGAATA**

**TCGACAGACTCAATTAGAAATATAGTGTCGTTGCAATATGTTATTCAATGCGATAAGGGT**

**GGCCCTTGCGGGGAAGACATCTCTCAAAAATGAGACCTCTATTGCAGTAGAGGTACAGGG**

**AGATGCCCATAAATTAAGAC**TCGAGCGGGATTTTATGGCTTTTTTAGGTATTTTTGTAAG

GGTAAAATAGGCCCATCAAACAGCATTAGAAATGCTAATCAGCCCAAAAAACAAAAGCAA

TCTTTTTTTGTTGCTAAAAGATAAAAATAAGTCGAGGCTGTGGTAACATATCCCACAGAT

TAAAGAAAGTCATAAGACTTGAATCTTCAGAATTTTAAAAAGCAGTTTTGCCAACGTAAG

ATTTTTGAAGTTTTCGACCAACAATACCGTTACTGGTATTTGTCTGTTAAAGATAAGCAT

TTTTGCTGGAGGAAAACCGCATGAGTAAAGGCGAAGAACTCTTTACCGGCGTGGTGCCCA

TTCTCGTGGAACTCGATGGCGATGTGAATGGCCACAAATTTAGTGTGAGTGGCGAAGGCG

AAGGCGATGCCACCTATGGCAAACTCACCCTCAAACTCCTCTGTACCACCGGCAAACTCC

CCGTGCCCTGGCCCACCCTCGTGACCACCCTCGGCTATGGCCTCCAATGTTTTGCCCGCT

ATCCCGATCACATGAAAAAACACGATTTTTTTAAAAGTGCCATGCCCGAAGGCTATGTGC

AAGAACGCACCATTTTTTTTAAAGATGATGGCAATTATAAAACCCGCGCCGAAGTGAAAT

TTGAAGGCGATACCCTCGTGAATCGCATTGAACTCAAAGGCATTGATTTTAAAGAAGATG

GCAATATTCTCGGCCACAAACTCGAATATAATTATAATAGTCACAATGTGTATATTACCG

CCGATAAACAAAAAAATGGCATTAAAGCCAATTTTAAAATTCGCCACAATATTGAAGATG

GCGGCGTGCAACTCGCCGATCACTATCAACAAAATACCCCCATTGGCGATGGCCCCGTGC

TCCTCCCCGATAATCACTATCTCAGTTATCAAAGTGCCCTCTTTAAAGATCCCAATGAAA

AACGCGATCACATGGTGCTCCTCGAATTTCTCACCGCCGCCGGCATTACCGAAGGCATGA

ATGAACTCTATAAATTATAATTTGAATCATGCGGAATGCGATCGCCTTAGGACGGTCGCA

TTTTTTGTTTACGTCTAAAATTAGTCGAAATCCCCCATCAACGCCATGGTAGTGATT*TTA*

*GACGTCGGAATTGCCAGCTGGGGCGCCCTCTGGTAAGGTTGGGAAGCCCTGCAAAGTAAA*

*CTGGATGGCTTTCTTGCCGCCAAGGATCTGATGGCGCAGGGGATCAAGATCTGATCAAGA*

*GACAGGATGAGGATCGTTTCGCATGATTGAACAAGATGGATTGCACGCAGGTTCTCCGGC*

*CGCTTGGGTGGAGAGGCTATTCGGCTATGACTGGGCACAACAGACAATCGGCTGCTCTGA*

*TGCCGCCGTGTTCCGGCTGTCAGCGCAGGGGCGCCCGGTTCTTTTTGTCAAGACCGACCT*

*GTCCGGTGCCCTGAATGAACTGCAGGACGAGGCAGCGCGGCTATCGTGGCTGGCCACGAC*

*GGGCGTTCCTTGCGCAGCTGTGCTCGACGTTGTCACTGAAGCGGGAAGGGACTGGCTGCT*

*ATTGGGCGAAGTGCCGGGGCAGGATCTCCTGTCATCTCACCTTGCTCCTGCCGAGAAAGT*

*ATCCATCATGGCTGATGCAATGCGGCGGCTGCATACGCTTGATCCGGCTACCTGCCCATT*

*CGACCACCAAGCGAAACATCGCATCGAGCGAGCACGTACTCGGATGGAAGCCGGTCTTGT*

*CGATCAGGATGATCTGGACGAAGAGCATCAGGGGCTCGCGCCAGCCGAACTGTTCGCCAG*

*GCTCAAGGCGCGCATGCCCGACGGCGAGGATCTCGTCGTGACCCATGGCGATGCCTGCTT*

*GCCGAATATCATGGTGGAAAATGGCCGCTTTTCTGGATTCATCGACTGTGGCCGGCTGGG*

*TGTGGCGGACCGCTATCAGGACATAGCGTTGGCTACCCGTGATATTGCTGAAGAGCTTGG*

*CGGCGAATGGGCTGACCGCTTCCTCGTGCTTTACGGTATCGCCGCTCCCGATTCGCAGCG*

*CATCGCCTTCTATCGCCTTCTTGACGAGTTCTTCTGAGCGGGACTCTGGGGTTCGA***cctt**

**gtgatgatcactaccgtttaaaagattgaaaaagaatatttttttgaccaataagtccag**

**tttattcaaaaaaaggcgctttgtatacctttaggaaaaagataatctcagcaaaaataa**

**gggcatttaggccagaaatgaagaacgtagaagtattttggcaagccagggatttcacct**

**aaaaattaacggttgataatccttctcaaaaataaaagaatgtccttgttagtccaggaa**

**accgaggtggcgatcgcctttggatcaagatctattcccagagttgcagaatttgtgccg**

**tgatgggatggcgtttcgatttttgtaacccggtgcgatggattggtcaggtgagtagcg**

**atggataatttttccggcctcttcaccccaaaaagatctagcggcttccctggggcatgg**

**gatgataggttaagctgtcttacggtcttttgcccctgatttTTCTATGGTTAGAATTAA**

**CGAAAATTACCTCAAA**

NS2_5’-P_rbc_-mOrange-T_rbc_-KmR-NS2_3’ (NS2 homology regions in bold, mOrange underlined, and KmR cassette in italics)

**TGGGGGCTTGTTGAAGATTTACTTTAGGCATTTACGCTTTAACTCCTTAAATCTAAAGAA**

**CGAAGAACTATATGGTGCGGCCCAGGGGGGCAGATACTTTACAAAACAATCATCATGTAG**

**GTCACAATCTAGAACACAAAGAATTTTTCTAAAAAATAAACGACATACCCTTTAACTTTC**

**TTACGCTTTATAACTTTTAATTGAGCGCTCACTACTTTAGACGGGCAACAATCGAAAAAG**

**GTTCAACGATTCATTAAAATCTCAGGGATGGGGAATTCATCGGCGTTCTATACTTAATTC**

**TCCTTGATTGACCAAGAGCTTGAAAAGGGGAAAAGATGCGGGATTACCGAACTTGGAATA**

**TCGACAGACTCAATTAGAAATATAGTGTCGTTGCAATATGTTATTCAATGCGATAAGGGT**

**GGCCCTTGCGGGGAAGACATCTCTCAAAAATGAGACCTCTATTGCAGTAGAGGTACAGGG**

**AGATGCCCATAAATTAAGAC**TCGAGCGGGATTTTATGGCTTTTTTAGGTATTTTTGTAAG

GGTAAAATAGGCCCATCAAACAGCATTAGAAATGCTAATCAGCCCAAAAAACAAAAGCAA

TCTTTTTTTGTTGCTAAAAGATAAAAATAAGTCGAGGCTGTGGTAACATATCCCACAGAT

TAAAGAAAGTCATAAGACTTGAATCTTCAGAATTTTAAAAAGCAGTTTTGCCAACGTAAG

ATTTTTGAAGTTTTCGACCAACAATACCGTTACTGGTATTTGTCTGTTAAAGATAAGCAT

TTTTGCTGGAGGAAAACCGCATGGTGAGTAAAGGCGAAGAAAATAATATGGCCATTATTA

AAGAATTTATGCGCTTTAAAGTGCGCATGGAAGGCAGTGTGAATGGCCACGAATTTGAAA

TTGAAGGCGAAGGCGAAGGCCGCCCCTATGAAGGCTTTCAAACCGCCAAACTCAAAGTGA

CCAAAGGCGGCCCCCTCCCCTTTGCCTGGGATATTCTCAGTCCCCAATTTACCTATGGCA

GTAAAGCCTATGTGAAACACCCCGCCGATATTCCCGATTATTTTAAACTCAGTTTTCCCG

AAGGCTTTAAATGGGAACGCGTGATGAATTTTGAAGATGGCGGCGTGGTGACCGTGACCC

AAGATAGTAGTCTCCAAGATGGCGAATTTATTTATAAAGTGAAACTCCGCGGCACCAATT

TTCCCAGTGATGGCCCCGTGATGCAAAAAAAAACCATGGGCTGGGAAGCCAGTAGTGAAC

GCATGTATCCCGAAGATGGCGCCCTCAAAGGCGAAATTAAAATGCGCCTCAAACTCAAAG

ATGGCGGCCACTATACCAGTGAAGTGAAAACCACCTATAAAGCCAAAAAACCCGTGCAAC

TCCCCGGCGCCTATATTGTGGGCATTAAACTCGATATTACCAGTCACAATGAAGATTATA

CCATTGTGGAACAATATGAACGCGCCGAAGGCCGCCACAGTACCGGCGGCATGGATGAAC

TCTATAAATTATAATTTGAATCATGCGGAATGCGATCGCCTTAGGACGGTCGCATTTTTT

GTTTACGTCTAAAATTAGTCGAAATCCCCCATCAACGCCATGGTAGTGATT*TTAGACGTC*

*GGAATTGCCAGCTGGGGCGCCCTCTGGTAAGGTTGGGAAGCCCTGCAAAGTAAACTGGAT*

*GGCTTTCTTGCCGCCAAGGATCTGATGGCGCAGGGGATCAAGATCTGATCAAGAGACAGG*

*ATGAGGATCGTTTCGCATGATTGAACAAGATGGATTGCACGCAGGTTCTCCGGCCGCTTG*

*GGTGGAGAGGCTATTCGGCTATGACTGGGCACAACAGACAATCGGCTGCTCTGATGCCGC*

*CGTGTTCCGGCTGTCAGCGCAGGGGCGCCCGGTTCTTTTTGTCAAGACCGACCTGTCCGG*

*TGCCCTGAATGAACTGCAGGACGAGGCAGCGCGGCTATCGTGGCTGGCCACGACGGGCGT*

*TCCTTGCGCAGCTGTGCTCGACGTTGTCACTGAAGCGGGAAGGGACTGGCTGCTATTGGG*

*CGAAGTGCCGGGGCAGGATCTCCTGTCATCTCACCTTGCTCCTGCCGAGAAAGTATCCAT*

*CATGGCTGATGCAATGCGGCGGCTGCATACGCTTGATCCGGCTACCTGCCCATTCGACCA*

*CCAAGCGAAACATCGCATCGAGCGAGCACGTACTCGGATGGAAGCCGGTCTTGTCGATCA*

*GGATGATCTGGACGAAGAGCATCAGGGGCTCGCGCCAGCCGAACTGTTCGCCAGGCTCAA*

*GGCGCGCATGCCCGACGGCGAGGATCTCGTCGTGACCCATGGCGATGCCTGCTTGCCGAA*

*TATCATGGTGGAAAATGGCCGCTTTTCTGGATTCATCGACTGTGGCCGGCTGGGTGTGGC*

*GGACCGCTATCAGGACATAGCGTTGGCTACCCGTGATATTGCTGAAGAGCTTGGCGGCGA*

*ATGGGCTGACCGCTTCCTCGTGCTTTACGGTATCGCCGCTCCCGATTCGCAGCGCATCGC*

*CTTCTATCGCCTTCTTGACGAGTTCTTCTGAGCGGGACTCTGGGGTTCGA***ccttgtgatg**

**atcactaccgtttaaaagattgaaaaagaatatttttttgaccaataagtccagtttatt**

**caaaaaaaggcgctttgtatacctttaggaaaaagataatctcagcaaaaataagggcat**

**ttaggccagaaatgaagaacgtagaagtattttggcaagccagggatttcacctaaaaat**

**taacggttgataatccttctcaaaaataaaagaatgtccttgttagtccaggaaaccgag**

**gtggcgatcgcctttggatcaagatctattcccagagttgcagaatttgtgccgtgatgg**

**gatggcgtttcgatttttgtaacccggtgcgatggattggtcaggtgagtagcgatggat**

**aatttttccggcctcttcaccccaaaaagatctagcggcttccctggggcatgggatgat**

**aggttaagctgtcttacggtcttttgcccctgatttTTCTATGGTTAGAATTAACGAAAA**

**TTACCTCAAA**

# Reference

Ruffing, A.M. (2014). Improved Free Fatty Acid Production in Cyanobacteria with Synechococcus sp. PCC 7002 as Host. *Frontiers in Bioengineering and Biotechnology* 2. doi: 10.3389/fbioe.2014.00017.
